# Supplementary material for: Cancer patients’ needs assessment in primary care: study protocol for a cluster randomised controlled trial (cRCT), economic evaluation and normalisation process theory evaluation of the needs assessment tool cancer (CANAssess)
Source: BMJ Open. 2022 May 4;12(5):e051394. doi: 10.1136/bmjopen-2021-051394 (PMC9073401; doi:10.1136/bmjopen-2021-051394)
Supplement: Supplementary data [file bmjopen-2021-051394supp001.pdf]

Supplementary file 1 –Risk of bias assessment

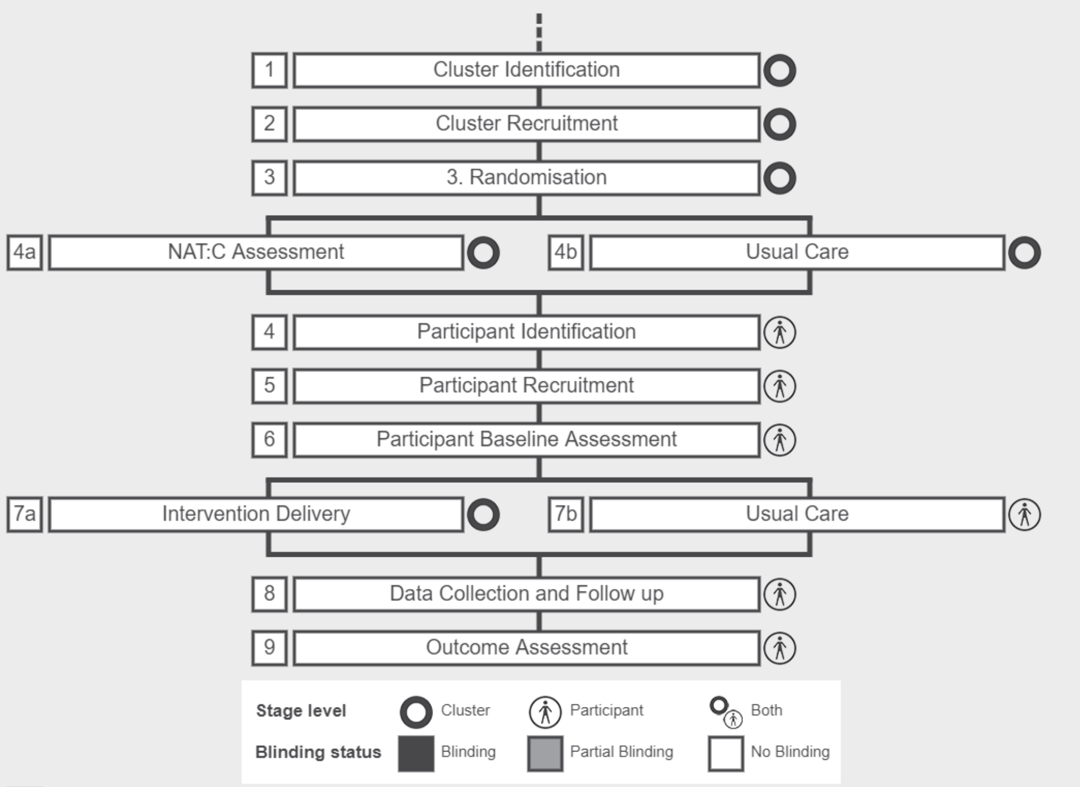

1. Cluster identification

General Practices in Yorkshire and Tyne and Wear will be approached and 54 recruited. Cluster identification will be conducted by four separate 'hubs', located at Leeds, Hull, Sheffield and the North East of England, each co-ordinated by a clinical 'hub-lead'. Practices will be invited to submit an Expression of Interest through relevant Clinical Research Network (CRN) mailing lists and hub lead networks. Practices will be asked to confirm their capacity to deliver the trial and eligibility will be assessed by the research team in terms of local research capacity.

2. Cluster recruitment

General Practices will be eligible unless they: took part in the feasibility study, have implemented or are planning to implement within the duration of the trial a systematic holistic cancer care intervention that overlaps with the NAT-C, or are unable to confirm capacity and capability to deliver the study at their GP Surgery. Practices will provide consent to deliver the study on the terms stated in a Schedule of Events Cost Attribution Template (SoECAT).

3. Randomisation

General practices will be randomised with a 1:1 ratio level by a statistician at the Leeds Clinical Trials Unit. Randomisation will take place post-site initiation. Practices will be randomised to either i) Needs Assessment Tool – Cancer NAT-C) plus Usual Care or ii) Usual Care, stratified by: Locality; Urban or rural area (UK government rural-urban classification based on GP Surgery postcode; List Size: <5000, 5000-10000, >10000 (obtained from NHS digital); A GP training practice (obtained from

site feasibility questionnaire): Yes, No. Practices will be randomised after consent and prior to study training and participant identification. Training will take place either face to face, via video-link or with a piloted online training package.

4. Participant Identification

An administrator or research nurse will conduct a database search for patients with 'active cancer' post-randomisation. A date restriction of five years will be applied in terms of date of diagnosis. This will remove historic cancer cases from the results, but may miss patients who have been living with active cancer for more than five years. A further exclusion will remove people with basal cell carcinoma (BCC) using a read code. A clinician will assess participant eligibility, in particular, to confirm a current cancer diagnosis and to confirm capacity to provide informed consent. There is a small risk that clinicians may exclude patients due to stage of illness. The research team will encourage clinicians to give patients at any stage of illness the opportunity to take part. Eligibility will be defined by a clinic and eligibility checks will take place during study monitoring conducted by a trained researcher.

5. Participant Recruitment

Eligible patients will be invited to the study either via letter, SMS or opportunistically at General Practices. All eligible patients will be provided with a Study Invitation Sheet. A Research Nurse will contact patients expressing interest in the study, answer any questions that the patient may and arrange informed written consent. Witnessed informed consent may be taken if a patient is unable to write.

Participating patients will be given the opportunity to nominate a carer if they would like to. Nominated carers will then receive a Carer Information Sheet and a Study Invitation. A Research Nurse will answer any questions that carer may have about the study ahead of arranging informed written consent.

Participants will find out which arm of the trial their practice has been allocated to after providing informed consent.

6. Participant Baseline Assessment

After taking written informed consent, a Research Nurse will help participants to provide baseline information. Demographic information and clinical characteristics will be collected. During a face to face appointment, a research nurse will collect participant: age, sex, cancer type and stage, treatment history, ethnicity, relationship status, living arrangement and accommodation, household income, postcode, the Australian Karnofsky Performance Status (AKPS) and the Charlson Co-morbidity Index. Patients will then be advised regarding their allocation and advised how to proceed win the study.

7a. Intervention Delivery

General Practitioners and clinical nurses will receive training in how to use the NAT:C either face to face or online. Research nurses will not receive intervention training. General practices will then contact patients to arrange a twenty minute needs assessment appointment, to occur within two weeks of informed consent. Clinicians will conduct a twenty-minute needs assessment appointment using the NAT-C. The NAT-C will be available as a template on EMIS and SystmOne and a paper copy will be available to clinicians if required. Patients may attend their needs assessment appointment

with a carer if they would like to. The carer does not have to be participating in the study. Patients will also have access their General Practice as usual.

7b. Usual Care

Patients will have access to their General Practice as usual.

8. Data collection and follow up

Patient participants will be asked to complete follow up questionnaires at one month and three months: the Supportive Care Needs Survey, the AKPS, the revised Edmonton Symptom Assessment System (ESAS-r), the EORTC QLQ-C15-PAL and a bespoke Resource Use Questionnaire (RUQ). Participants will be supported by a research nurse during data collection either face to face or over the phone. Completed NAT:C assessments will be retrieved from the practice clinical record. It will not be possible to blind research nurses to the allocation of General Practices (and therefore patients) during data collection. However, data collection will not be conducted by anybody who has been involved in delivering the intervention. Data collection will be undertaken as close to the stated time points as feasible.

9. Outcome assessment

Primary outcome will be proportion of patients with an unmet need on the SCNS. Analysis will be conducted on an intent to treat basis. Final analysis will be conducted by a senior statistician at the Leeds Clinical Trials Unit and will take place once all participants have completed three month measures.
