# Supplementary material for: Cancer patients’ needs assessment in primary care: study protocol for a cluster randomised controlled trial (cRCT), economic evaluation and normalisation process theory evaluation of the needs assessment tool cancer (CANAssess)
Source: BMJ Open. 2022 May 4;12(5):e051394. doi: 10.1136/bmjopen-2021-051394 (PMC9073401; doi:10.1136/bmjopen-2021-051394)
Supplement: Supplementary data [file bmjopen-2021-051394supp002.pdf]

Delete this line, then print first page on practice-headed paper

CANAssess
Cancer Patients' Needs Assessment in Primary Care

Funded by yorkshire cancer research

CANAssess 2: Cancer Patients' Needs Assessment in Primary Care – A Cluster Randomised Controlled Trial

Participant ID:	Initials:
Date of Birth:	NHS Number:
ISRCTN: 15497400	Principal Investigator:

PARTICIPANT CONSENT FORM

	*Please **initial** each box below*
1. I confirm that I have read and understand the information sheet dated <<INSERT DATE>> (version X.0) for the above study. I have had the opportunity to consider the information, ask questions, and have had these answered satisfactorily	
2. I understand that my participation in this study is voluntary and that I am free to withdraw at any time without my medical care or legal rights being affected.	
3. I understand that if I withdraw from the above study, the data collected from me up until that point will be used in analysing the results of the study.	
4. I agree for my personal details (including name, date of birth, address, postcode, email address, telephone number, NHS number, GP name and GP address) to be securely stored in accordance with the study sponsor guidance (minimum 5 years).	
5. I understand that relevant sections of any of my medical records and/or study data may be looked at by responsible individuals from the research team, the sponsor (University of Hull), Leeds Clinical Trials Research Unit (CTRU), relevant third parties or from regulatory authorities where it is relevant to my taking part in the research. I give permission for these individuals to access my records.	
6. I understand that if during this study my clinical care team determine that I have lost my ability to make my own decisions, I will be withdrawn from the study and no further study information will be collected. The data collected from me up until that point will be used in analysing the results of the study.	
7. I consent to the secure transfer, storage and use of paper and electronic personal information, for the purposes of this study to the CTRU, or relevant third parties. I understand that any information that could identify me will be kept strictly confidential and that no personal information will be included in the study report or other publication.	
8. I agree to a copy of this Consent Form being sent to the CTRU.	

Clark J, *et al. BMJ Open* 2022; 12:e051394. doi: 10.1136/bmjopen-2021-051394

Delete this line, then print first page on practice-headed paper

9. I agree to my General Practitioner being informed of my participation in this study and being provided with a copy of this consent form. I understand that my GP will be advised of any significant information relating to my health that comes to light.	
10. I agree to take part in the study.	

Optional:

Even if you agree to take part in this study, you do not have to agree to this statement. Please initial next to 'yes' **or** 'no'.

11. I agree that the information collected about me may be used to support other research in the future, and may be shared anonymously with other researchers.	Yes	
	No	

Method of Consent:

☐ **Telephone Consent:**

Researcher:

I have explained the study and read each consent statement to the above named patient. He/she has indicated his/her willingness to participate and agreed to each compulsory statement, so I have initialled and signed on their behalf.

Signature……………………………………..……………………………………

Name (block capitals)……………………………………………….…………………

Date…………………………………………………….………………………………

☐ **Face-To-Face Consent:**

Patient:

Signature…………………………………………………………………………………

Name (block capitals)…………………………………………………….………………

Date……………………………………………………….……………………………

Clark J, *et al. BMJ Open* 2022; 12:e051394. doi: 10.1136/bmjopen-2021-051394

Delete this line, then print first page on practice-headed paper

Researcher:

I have explained the study to the above named patient and he/she has indicated his/her willingness to participate.

Signature…………………………………………..…………………………………

Name (block capitals)…………………………………………………..……………………

Date…………………………………………………………….………………………………

Witness/Translator:

I have completed this consent form on behalf of the person named above who has freely given their verbal consent to participate.

Signature…………………………………………..…………………………………

Name (block capitals)……………………………………………………………………

Date…………………………………………………………….………………………………

(1 copy for patient; 1 for the CTRU; 1 held in patient notes, original stored in Investigator Site File)

Clark J, *et al. BMJ Open* 2022; 12:e051394. doi: 10.1136/bmjopen-2021-051394
