# Supplementary material for: Cancer patients’ needs assessment in primary care: study protocol for a cluster randomised controlled trial (cRCT), economic evaluation and normalisation process theory evaluation of the needs assessment tool cancer (CANAssess)
Source: BMJ Open. 2022 May 4;12(5):e051394. doi: 10.1136/bmjopen-2021-051394 (PMC9073401; doi:10.1136/bmjopen-2021-051394)
Supplement: Author's manuscript [file bmjopen-2021-051394.draft_revisions.pdf]

BMJ Open is committed to open peer review. As part of this commitment we make the peer review history of every article we publish publicly available.

When an article is published we post the peer reviewers' comments and the authors' responses online. We also post the versions of the paper that were used during peer review. These are the versions that the peer review comments apply to.

The versions of the paper that follow are the versions that were submitted during the peer review process. They are not the versions of record or the final published versions. They should not be cited or distributed as the published version of this manuscript.

BMJ Open is an open access journal and the full, final, typeset and author-corrected version of record of the manuscript is available on our site with no access controls, subscription charges or pay-per-view fees (http://bmjopen.bmj.com).

If you have any questions on BMJ Open's open peer review process please email info.bmjopen@bmj.com

BMJ Open

CANcer Patients' Needs Assessment in Primary Care: Study Protocol for a Cluster Randomised Controlled Trial (cRCT), economic evaluation and Normalisation Process Theory evaluation of the Needs Assessment Tool Cancer (CANAssess)

Journal:	*BMJ Open*
Manuscript ID	bmjopen-2021-051394
Article Type:	Protocol
Date Submitted by the Author:	18-Mar-2021

Complete List of Authors:	Clark, Joseph; University of Hull, Wolfson Palliative Care Research Centre<br>Copsey, Bethan; University of Leeds Clinical Trials Research Unit,<br>Wright-Hughes, Alexandra; University of Leeds, Clinical Trials Research Unit<br>McNaught, Emma; University of Leeds, Clinical Trials Research Unit<br>Bijsterveld, Petra; University of Leeds, Clinical Trials Research Unit<br>McCormack, Terry; University of Hull, Hull York Medical School<br>Foy, Robbie; University of Leeds, Leeds Institute of Health Sciences<br>Wilkes, Scott; University of Sunderland, School of Pharmacy<br>Dickson, Jon; The University of Sheffield, Academic Unit of Primary Medical Care<br>Meads, David; University of Leeds, Leeds Institute of Health Sciences<br>Farrin, Amanda; University of Leeds, Clinical Trials Research Unit<br>Johnson, Miriam; The University of Hull, Wolfson Palliative Care Research Centre

Keywords:	PALLIATIVE CARE, PRIMARY CARE, Adult palliative care < PALLIATIVE CARE

SCHOLARONE™
Manuscripts

Title page

CANcer Patients' Needs Assessment in Primary Care: Study Protocol for a Cluster Randomised Controlled Trial (cRCT), economic evaluation and Normalisation Process Theory evaluation of the Needs Assessment Tool Cancer (CANAssess)

Authors

Dr Joseph Clark, Wolfson Palliative Care Research Centre, University of Hull, United Kingdom

Dr Bethan Copsey, Leeds Institute of Clinical Trials Research, University of Leeds, United Kingdom

Dr Alexandra Wright-Hughes, Leeds Institute of Clinical Trials Research, University of Leeds, United Kingdom

Ms Emma McNaught, Leeds Institute of Clinical Trials Research, University of Leeds, United Kingdom

Ms Petra Bijsterveld, Leeds Institute of Clinical Trials Research, University of Leeds, United Kingdom

Dr Terry McCormack, Hull York Medical School, University of Hull, United Kingdom

Professor Robbie Foy, University of Leeds, United Kingdom

Professor Scott Wilkes, University of Sunderland, United Kingdom

Dr Jon Dickson, University of Sheffield, United Kingdom

Dr David Meads, University of Leeds, United Kingdom

Professor Amanda Farrin, Leeds Institute of Clinical Trials Research, University of Leeds, United Kingdom

Professor Miriam Johnson, Wolfson Palliative Care Research Centre, University of Hull, United Kingdom

Corresponding author

Dr Joseph Clark, Wolfson Palliative Care Research Centre, University of Hull, Cottingham Road, Hull, United Kingdom, HU6 7RX

E: joseph.clark@hyms.ac.uk

T: 01482 347538

F: N/A

Key words

Palliative Care, Primary Health Care, Cancer Care, Cluster Randomised Trial, Economic Evaluation, Normalisation Process Theory

Word count

3954

For peer review only

Abstract

Introduction: Unmet needs in patients with cancer and their carers are common but poorly identified and addressed. The Needs Assessment Tool-Cancer (NAT-C) is a structured consultation guide to identify and triage patient and carer unmet needs. The NAT-C is validated, but its effectiveness in reducing unmet patient and carer needs in primary care is unknown.

Methods and analysis: Cluster randomised controlled trial with internal pilot and embedded process evaluation to test the clinical and cost effectiveness of the NAT-C in primary care for people with active cancer in reducing unmet patient and carer need, compared with usual care. We will recruit 1080 patients with active cancer (and carers if relevant) from 54 general practices in England.

Participating practices will be randomised 1:1 to either deliver a NAT-guided clinical consultation plus usual care or to usual care alone. Consenting participants with active cancer and their carers (if nominated) will be asked to complete study questionnaires at baseline, one and three months for all, six months except for those recruited outside of the last three months of recruitment, and attend a NAT-C appointment if allocated to an intervention practice. An internal pilot will assess: site and participant recruitment, intervention uptake, and follow-up rates. The primary outcome, the proportion of patients with an unmet need on the Supportive Care Needs Survey Short Form 34 (SCNS-SF34) at three months post registration, will be analysed using a multi-level logistic regression. Mixed-methods process evaluation informed by Normalisation Process Theory will use quantitative survey and interview data from clinicians and key stakeholders in cancer care to develop an implementation strategy for nationwide rollout of the NAT-C if the intervention is cost-effective.

Ethics and dissemination: If effective, the NAT-C will become the gold standard for cancer primary care. REC code: 20/LO/0312

Trial registration: ISRCTN15497400, registered 07/04/2020

For peer review only

For peer review only - http://bmjopen.bmj.com/site/about/guidelines.xhtml

Strengths and limitations

- We are testing the clinical and cost effectiveness of the Needs Assessment Tool – Cancer (NAT-C) which has been validated and shown to be acceptable to patients and clinicians during feasibility testing.
- Feasibility testing led to modifications of intervention delivery and informed trial design, making successful completion of the trial more likely.
- CANASSESS is a cluster randomised controlled trial of 54 general practices across two regions in England, making it likely that findings will be generalizable nationwide.
- By necessity, participants, health professionals delivering the intervention and study researchers will be aware of treatment allocation; potential bias will be monitored during the trial.
- COVID-19 presents unique challenges in terms of safely conducting clinical trials in primary care.

For peer review only

For peer review only - http://bmjopen.bmj.com/site/about/guidelines.xhtml

Introduction

Unmet needs in people with cancer and their carers are common but poorly identified and addressed. Many people with cancer experience unmet needs across multiple domains.[1] General Practitioners (GPs, family doctors) and other clinicians in primary care would like to do more to support their cancer patients, but there is no agreed evidence-based best approach.[2] Difficulties are compounded by inconsistent co-ordination of care with oncology services as GPs may be unaware of problems unless patients present directly. However, people with cancer often do not attend primary care for cancer care and systematic, routine holistic assessment of patient problems is rare.[3] In addition, patients commonly volunteer only the most pressing problem to their clinicians; open enquiry in one study only found an average of one problem presented, whereas systematic enquiry discovered an average of ten, many of which were severe and distressing.[4]

Tools are available to assist clinicians caring for people with cancer,[5] but few are designed to identify and triage care needs in the everyday busy clinical setting and across all stages of active disease from diagnosis through to end of life care. Furthermore, although needs assessment tools are advocated,[6] there is no rigorous research evidence to indicate whether they actually improve practice and patient outcomes. A needs assessment tool can reduce unmet needs by providing a consistent and comprehensive approach to prompting discussion patients' range of support and care needs; helps professionals triage tailored action and is useful for audit and service planning.[7,8,9,10,11] Through triage, an assessment tool may help reduce late referrals for palliative care, and improve referrals where there are physical, psychological, social and spiritual problems.[12, 13] However, tools currently available are commonly highly detailed and long for daily clinical use.[14,15,16]

Development of the Needs Assessment Tool Cancer

The Needs Assessment Tool – Cancer (NAT-C) was developed in Australia, where it has been shown to reduce unmet needs of patients in oncology clinics.[3] We adapted and validated this tool for use in UK primary care.[17] Use of the NAT-C aims to reduce unmet supportive and palliative care needs of cancer patients and their carers by supporting systematic clinician assessment of patient and carer needs across multiple domains. Identified problems may be managed in primary care or through referral to other services.

Our Phase II feasibility study found that a randomised trial is feasible in terms of recruitment, data quality, and intervention delivery.[18] Required changes to improve study processes were identified, specifically, confirmation of participant acceptability to be directed to a known NAT-C clinician. Clinicians, patients and carers also viewed the tool positively and supported need for a definitive trial. A key alteration to the NAT-C was to develop the paper-based tool into digital templates for use in standard electronic clinical record systems (EMIS, SystmOne) in accordance with clinician preferences.

Aims

The CANAssess trial aims to evaluate the clinical effectiveness and cost effectiveness of the NAT-C in reducing unmet needs of patients and carers in primary care carer compared to usual care alone.

Methods and analysis

Design summary

For peer review only - http://bmjopen.bmj.com/site/about/guidelines.xhtml

CANAssess is a multicentre, two-arm, pragmatic, cluster randomised controlled trial (cRCT) with 12-month internal pilot, embedded process evaluation and cost-effectiveness evaluation. A cRCT design reflects that the intervention would be implemented at general practice level and reduces contamination in the control group.

Trial objectives and outcomes

Box 1: CANAssess Primary, Secondary, internal pilot, economic and process evaluation objectives

Primary Objective: To test the effectiveness of the NAT-C compared to usual care in reducing unmet patient need as measured using the Supportive Care Needs Survey Short Form 34 (SCNS-SF34[19]) at three months post registration.

Secondary objectives:

To evaluate the effectiveness of the NAT-C compared to usual care with regard to:

- Patient unmet need on psychological, health system information, physical and daily activity, patient care and support, and sexuality domains of the SCNS-SF34 at one, three and six months;
- Patient performance status, measured using the Australian-modified Karnofsky Performance Status (AKPS[20]) at one, three and six months;
- Patient severity of symptoms, measured using the Revised Edmonton Symptom Assessment System (ESAS-r[21]) at one, three and six months;
- Patient mood and quality of life as measured by the European Organisation for Research and Treatment of Cancer Quality of Life-C15-Palliative questionnaire (EORTC QLQ-C15-PAL[22]) at one, three and six months; and
- Carers' ability to care and carer wellbeing as measured using the Carer Experience Scale (CES[23]) and Zarit Burden Interview-12 (ZBI[24]) at one, three and six months.

To evaluate intervention delivery, uptake and fidelity of the NAT-C as measured by:

- NAT-C training of General Practitioners (GPs) and nurses in each general practice;
- Completed NAT-C consultations by patient and general practice (including completion of individual items of the NAT-C);
- Length of NAT-C consultations; and
- Referral patterns and actions taken to meet identified unmet need (including referrals to health professionals and/or services) from the completed NAT-C.

INTERNAL PILOT objectives:
To assess sufficiency of numbers of general practices and patients at 12 months post start of recruitment; we will proceed with the trial unchanged if we have 80% (43) sites open and are recruiting to 80% (48 participants per month) of target. We will assess intervention uptake, follow-up rates, and potential for selection bias.

HEALTH ECONOMIC objectives:
Service utilisation, referral patterns and cost-effectiveness measured using:

- Bespoke Resource Use Questionnaire (RUQ) for capturing patient healthcare service utilisation and referral patterns at one, three and six months; and
- The EQ-5D-5L[25], ICEpop CAPability Supportive Care Measure (ICECAP-SCM[26]) and CES to generate Quality-Adjusted Life Years (QALYs) and estimates of well-being at one, three and six months.

For peer review only - http://bmjopen.bmj.com/site/about/guidelines.xhtml

PROCESS EVALUATION objectives
To assess the adequacy of NAT-C training, intervention fidelity, possible mechanisms of action and issues regarding implementation in practice if the intervention is effective.

Recruitment setting

The study aims to recruit patients and their carers from 54 general practices (clusters) from 4 geographical regions (recruitment “hubs”) in Yorkshire and the North East of England. Locations were selected to ensure a range of multi-ethnic, rural and urban populations to maximise generalisability of findings.

Recruitment of general practices

Site identification and recruitment is detailed in Figure 1. General practices will be eligible unless they: took part in the feasibility study, have or are planning to implement within the duration of the trial a systematic holistic cancer care intervention that overlaps with the NAT-C, or lack capacity and capability to deliver the study.

Figure 1: Study Flow Chart

Cluster Randomisation

Where practice manager agreement is obtained, capacity and capability confirmed, and initial read-code search completed, participating general practices (clusters, n=54) will be randomised sequentially via an automated system at the Clinical Trials Research Unit (CTRU). General practice randomisation will be 1:1 to: implement the NAT-C in addition to usual care, or usual care alone, using a computer-generated minimisation programme incorporating a random element to ensure arms are balanced for stratification factors:

- Locality: Urban or rural area.[27, 28]
- List Size: <5000, 5000-10000, >10000[28]
- A GP training practice (obtained from site feasibility questionnaire): Yes, No

General practices and research nurses providing participant recruitment and follow-up support across multiple surgeries will, by necessity, be aware of treatment allocation. However, no member of the research team will be involved with intervention delivery to minimise performance bias. A structured risk of bias assessment is presented in Supplementary File 1. Participating practices will be free to withdraw from the study without negative consequence. In the event of practice withdrawal, we will inquire about reasons for withdrawal and may recruit replacement practices.

Participant eligibility

Eligibility criteria are shown in Box 2

Box 2: Patient/Carer Inclusion/Exclusion

Patient Inclusion Criteria
- Adults (aged 18 years and above)
- Diagnosis of active cancer (receiving anti-cancer treatment both with curative or palliative intent; managed with “watch and wait”; recurrent or metastatic; or inoperable)
- Willing and able to complete questionnaires at the trial follow-up schedule
- Provision of written or observed verbal informed consent.

For peer review only - http://bmjopen.bmj.com/site/about/guidelines.xhtml

For peer review only

- Sufficient knowledge of the English language to provide informed consent and complete trial questionnaires. The use of an appropriate translator/interpreter is allowed.

Patient Exclusion Criteria

- Patients in complete remission (no clinical or radiological evidence of cancer, and at least one month post anti-cancer treatments)
- Patients with basal cell carcinoma
- Patients living in a care home or other institutional setting
- Patients within one month of receiving their initial cancer diagnosis.

Carer Inclusion Criteria

- Adults (aged 18 and above)
- Nominated by participant
- Able to complete trial measures
- Written or observed verbal informed consent.
- Sufficient knowledge of the English language to provide informed consent and complete trial questionnaires. The use of an appropriate translator/interpreter is allowed.

Carer Exclusion Criteria

- Employed to look after the participant.

Participant recruitment

General practices will identify eligible patients by searching cancer registers and screening for eligibility. Eligible patients will be sent a letter with a Patient Information Sheet and expression of interest form. General practices may also send an SMS text message or amended letter to patients inviting them to express interest in the study on the CANAssess website. Consented patients may nominate carers for participation in the trial. Carers agreeing to participate will provide consent. The full process of participant recruitment is presented in Figure 2. For any participant or carer who wishes to withdraw from the trial, we will collect a reason for withdrawal and cease data collection, but keep collected data unless otherwise requested.

Figure 2. Participant and carer recruitment

Intervention Arm (NAT-C plus Usual Care)

The NAT-C comprises five sections: priority referral for further assessment, patient wellbeing, ability of carer or family to care for patient, carer/family wellbeing and resulting referrals (if required). Clinicians will be encouraged to use the tool as an *aide memoire*, conducting a holistic patient assessment as usual, but referring to the NAT-C to ensure all domains are addressed during a consultation. The NAT-C will be completed using either the electronic medical record template (EMIS, SystmOne) or on paper. Completed paper copies of the NAT-C will be uploaded to the patient record.

At least two clinicians per practice will be trained to use the NAT-C either face to face, via webinar or online using a training package piloted during feasibility work.

Participating patients at intervention arm surgeries will be offered a 20 minute appointment or home visit depending on clinical need, guided by a NAT-C trained clinician using the tool within approximately two weeks of study registration. Appointments will take place either at the practice,

For peer review only - http://bmjopen.bmj.com/site/about/guidelines.xhtml

at patients' homes or remotely via phone or video according to clinical judgement and coronavirus guidelines. Participating carers will be welcome to accompany patients to their appointment, however, the NAT-C allows assessment of carer need through patient response.

Usual care

Usual care is defined as management normally provided for patients with cancer registered at the general practice concerned.[29]

Data collection

Required data, assessment tools, collection time points and processes are summarised in Table 1.

Table 1: Summary of assessments[a]

Participant Assessment (including who is involved)	TIMELINE (months post-randomisation)			
	Baseline	1	3	6
**Eligibility and Consent**				
Consent (P, C, R)	X			
Eligibility (assessed by clinician, R)	X			
**Background and demographics**				
General Demographics (P, C, R)	X			
Cancer Demographics (R - case notes)	X			
Co-morbidities (R - case notes)	X			
**Follow-up data** (collected from case notes)				
Survival status (R)	Ongoing and at the overall end of the trial			
Related Unexpected Serious Adverse Events (R)	Ongoing			
NAT-C Intervention (R)	One month post participant registration			
Usual Care Data (R)	X	X	X	X
**Pre-questionnaire** (phone call at 1, 3, 6 months)				
Performance status (AKPS)	X	X	X	X
COVID status	X	X	X	X
**Participant Questionnaire Booklet** (Self-Completion with researcher support if needed)				
Unmet needs (SCNS-SF34)	X	X	X	X
Symptoms (ESAS-r)	X	X	X	X
Mood and Quality of Life (EORTC QLQ-C15-PAL)	X	X	X	X
EQ-5D-5L	X	X	X	X
ICECAP-SCM	X	X	X	X
Health Care Resource Use (including usual care data and referrals)	X	X	X	X
**Carer Questionnaire Booklet** (Self-Completion with researcher support if needed)				
Carer Experience Scale (CES)	X	X	X	X
Carer wellbeing and burden (ZBI-12)	X	X	X	X

[a] P, participant; C, carer-giver; R, researcher.

Baseline assessments

Clinical data including co-morbidities, cancer stage and treatments will be collected at baseline by the research nurse from the participant's medical record. Demographic information will be collected

For peer review only - http://bmjopen.bmj.com/site/about/guidelines.xhtml

on participants, including age, sex, participant ethnicity, and living arrangements, during the researcher baseline discussion. For carers, age, sex, relationship status and living arrangements will be collected.

Participant questionnaires

Self-reported participant and carer outcome measures will be collected via questionnaires at baseline, one month and three months post-registration. Questionnaires will also be collected at six months for participants and carers registered before 3 months prior to the end of participant recruitment.

Participants will be able to complete questionnaires using paper forms sent by post, online via REDCap or with a researcher over the phone or face-to-face, as appropriate. Only CTRU data and statistical staff will have direct access to the dataset.

Researchers will telephone participants to confirm questionnaire receipt and assess and collect Performance (AKPS) and COVID-19 status.

Intervention data collection

A research nurse will collect information on NAT-C intervention delivery and content, including the timing, duration, mode of delivery, referrals and subsequent appointments from the participant's medical record.

Safety data collection

In this population, it is expected that episodes of acute illness, infection, new medical problems and deterioration of existing medical problems will occur and could result in prolonged hospitalisation, hospital re-admission, significant or permanent disability or incapacity, or death.

Only serious adverse events fulfilling the definition of a Related Unexpected Serious Adverse Event (RUSAE) resulting from administration of any research procedure, and participant deaths during the trial period, will be recorded. Survival status of participants will be ascertained by research nurses from general practices ahead of sending study follow-up questionnaires.

Deaths

The date and cause of all deaths occurring during the trial period (to last participants 3 month follow-up assessment) will be collected by the researcher from participant's medical record.

STATISTICAL CONSIDERATIONS

Sample Size

The study has been powered to detect improvement in patients' level of unmet need as measured by proportion of patients reporting at least one moderate or high need in domains of the SCNS-SF34.[30]

Assuming that the proportion of patients with an unmet need on any SCNS-SF34 domain will be similar to that observed pre-intervention by Waller 2012[3]: 64%, then a sample size of 1080 patients recruited from approximately 54 general practices (540 patients, 27 practices per arm), will provide 85% power with a 5% significance level to detect a relative difference of 22% in the proportion of patients with an unmet need. This is an absolute difference of 14%, from 64% to 50%.

For peer review only - http://bmjopen.bmj.com/site/about/guidelines.xhtml

The sample size assumes: a 20% loss to follow up rate by 3 months, to account for eligible patients who are, or are nearing, end of life; an Intra-cluster Correlation Coefficient (ICC) of 0.05; an average general practice size of 20; and an adjustment to account for variable practice sizes of 4-40. Given heterogeneity in the design of palliative care services and availability of resources through general practices, and median ICCs reported for outcome variables (0.03) and primary care settings (0.045), an ICC of 0.05 will be used.[31]

Internal pilot and progression criteria

The internal pilot will end 12 months from recruitment of the first general practice. Data from participants in the internal pilot will be included in the main study analysis.

Progression criteria for recruitment are shown in Table 2, based on a traffic-light system of green (go), amber (review) and red (stop), and has been agreed by an independent Trial Steering Committee (TSC) and funder. The TSC will be provided with descriptive data, presented by arm and by general practice to assess internal pilot progression criteria, adherence to the intervention and follow-up, and selection bias at approximately 12 months after the start of the recruitment to inform a decision on continuation of the trial.

Table 2. Progression criteria for internal pilot

Criteria	Green (go)	Amber (review)	Red (stop)
**Recruitment**<br>General practices assessed at 12 months	80% open (≥43)	50% to 80% open (27-42)	<50% open (<27)
**Recruitment**<br>Participants per month assessed at 12 months (target after 3 months: 60pm)	≥80% (≥48)	50% to 80% (30-47)	<50% (<30)

Statistical analysis

There are no planned interim analyses; outcome data will be analysed once only. All analyses will be conducted on the Intent-to-Treat (ITT) population, in which all general practices and participants will be included in the analysis according to the group which the GP practice was randomised, and regardless of non-adherence to the intervention or withdrawal from the study. A two-sided 5% significance level will be used for statistical endpoint comparisons.

The flow of patients and general practices through the trial will be presented in a Consolidated Standards of Reporting Trials (CONSORT) diagram.

As appropriate for cluster trials recruiting participants after randomisation[32], statistical testing of baseline participant data will be at the end of the internal pilot and at the end of the study to assess for selection bias.

Analyses of primary (overall unmet need) and secondary outcomes (unmet needs, severity of symptoms, quality of life, carer wellbeing and burden) will use multi-level logistic or linear regression

For peer review only - http://bmjopen.bmj.com/site/about/guidelines.xhtml

(as appropriate) with participants nested within general practices, and general practices treated as a random effect. The model will be adjusted for the following fixed effects: GP practice-level stratification factors, important participant-level covariates (e.g. baseline unmet need, age, sex, cancer status, baseline performance status), and other relevant known predictors of outcome. Results will be expressed as point estimates, p-values, ICCs and 95% confidence intervals.

Reasons for attrition and missing participant data will be summarised and mechanisms for missing data we explored according to participant characteristics, intervention and control groups.[33] To conduct analysis on the ITT population, missing data will be multiply imputed at individual participant level under the missing at random assumption. Sensitivity analyses of the primary endpoint will be conducted to assess impact of missing data, choice of imputation model and missing at random assumption.

Quantitative summaries for AKPS score and corresponding change from baseline will be presented at baseline and month 1, 3, and 6 by treatment group. Intervention delivery will be summarised overall and by general practice to evaluate uptake of the NAT-C, adherence to the processes and quality of intervention delivery.

ECONOMIC EVALUATION

Within-trial health economic evaluation will be undertaken to assess cost-effectiveness of NAT-C vs. usual care. The cost-utility analysis will be conducted alongside the trial and follow National Institute for Health and Clinical Excellence (NICE) reference case for health technology appraisals.[34] The main health outcome will be QALYs based on the EQ-5D-5L (base case). Supplementary analyses will estimate cost per improvement in ICECAP-SCM and CES.

We will fully cost intervention delivery and measure service utilisation using a bespoke Resource Use Questionnaire (RUQ) and measure outcomes using the EQ-5D-5L, ICEpop CAPability Supportive Care Measure (ICECAP-SCM) and CES at one, three and six months.

A patient-completed RUQ will gather data on community-based (e.g. contact with GPs, nurses and physiotherapists/occupational therapists), specialist palliative care (hospice, hospital or community) and hospital-based (e.g. A&E visits and hospital attendances) healthcare resource utilisation at follow-up. Participants will be given a diary planner to keep to note any health care attendances to facilitate completion of the RUQ. Costs will be estimated using UK NHS reference unit costs, data from the Personal Social Services Research Unit (PSSRU) and British National Formulary. The primary perspective is the health and personal social service provider but a secondary analysis will adopt a wider perspective to incorporate costs and productivity loss incurred by patients and carers.

Results will be presented as incremental cost effectiveness ratio (ICERs). Results will also be presented as expected net monetary benefit and cost effectiveness acceptability curves (CEAC) based on non-parametric bootstrapping.[35] The analysis will employ regression models to adjust for baseline imbalances and account for the correlation between costs and QALYs.[36] The analysis will assume a willingness to pay threshold of £20,000 per incremental QALY with ICERs below this value indicating cost effectiveness.

PROCESS EVALUATION

A mixed-methods sub-study will use Normalisation Process Theory (NPT) to structure data collection and analysis of: 1) implementation of the NAT-C in trial general practices 2) clinicians' and staff

For peer review only - http://bmjopen.bmj.com/site/about/guidelines.xhtml

perspectives on the usefulness and effectiveness of the NAT-C, how this relates to usual care and how, if effective, the NAT-C could be implemented nationwide.

NPT is a well-established framework for understanding the dynamics involved in implementing, embedding and integrating a new intervention. We will draw upon quantitative and qualitative elements to identify issues related to implementation in terms of 1) a quantitative NPT survey to elicit the views of clinicians who have undergone NAT-C training and 2) qualitative interviews/focus groups with general practice staff, clinicians and external stakeholders with key roles in health policy and commissioning, relevant to cancer care in primary care.

Normalization MeAsure Development Questionnaire (NoMAD) survey

The NPT survey (NoMAD instrument) is a 23-item instrument for measuring implementation processes from the perspective of professionals directly involved in the work of implementing complex interventions. During feasibility testing, we adapted the NoMAD instrument in to a 17-point checklist to specifically address the NAT-C. Clinicians will be invited to complete the NoMAD survey either on paper or online following completion of NAT-C training (Survey 1). Using results from Survey 1, emerging qualitative findings and experiences, the NoMAD will be adapted to include questions regarding emerging issues and concerns. At the end of a practices' involvement with the study, clinicians who have used the NAT-C will be asked to complete the adapted NoMAD survey (Survey 2).

Clinicians will be asked questions on a Likert scale in relation to issues such as: attitudes to the NAT-C, NAT-C training and implementation concerns. Completion of the survey will imply informed consent. Data collection and management for surveys 1 and 2 will be delivered by the University of Hull. All survey data will be anonymised.

Interviews and focus groups

Opinion regarding NAT-C training, the role and place of the NAT-C within routine practice will be sought from clinicians who received NAT-C training and experts from a range of stakeholder groups (e.g. local commissioning groups, general practice federations, the National Cancer Research Institute's primary care group, Royal College of GPs, and Macmillan). Semi-structured interviews and focus groups using *a priori* topic guides (either phone/video conferencing or face-to-face, as appropriate) will be conducted at various time-points post-NAT-C use and up to the end of study. Interviews/focus groups with clinicians and key stakeholders will focus upon structural and policy issues relevant to potential implementation of the NAT-C in general practices nationwide, should trial results be positive.

Maximum variation purposive sampling will be used to optimise exploration of a range of clinicians, practice staff and key stakeholder perspectives. An initial purposive sampling grid for clinicians (profession, years of clinical practice, randomisation strata) will be expanded with further criteria identified from implementation study survey responses.

A sample of 15-20 clinicians and general practice staff and 10 -15 experts from a range of stakeholders will be sought through interviews or focus group.

Potential interviewees will be provided with a Study Invitation, a Study Information Sheet and asked to provide informed written consent prior to study procedures. All interviews and focus group discussions will be audio-recorded.

For peer review only - http://bmjopen.bmj.com/site/about/guidelines.xhtml

NoMAD Survey analysis

Free-text responses in survey 1 will be monitored by the implementation study researcher to enable rapid feedback to inform subsequent training at other sites.[37]

Once all surveys 1 and 2 are completed, free text responses will be subject to thematic analysis and descriptive statistics will be used to analyse Likert scale responses including: 1) the extent to which the intervention fits with current practice in relation to the components of NPT; 2) the potential relevance of the NAT-C to individuals' roles; 3) adequacy of NAT-C training; and 4) clinician attitudes to the NAT-C at baseline and at the end of the trial from Survey 1 and 2.

Interview/focus group analysis

Qualitative data will be analysed using thematic analysis[37], informed by NPT, relating to: how clinicians understand the intervention (coherence); how they engage with it (cognitive participation); enact it (collective action); and appraise its effects (reflexive monitoring).[38] The end of trial analysis will develop themes in relation to how the NAT-C could be implemented in primary care nationally, should trial be results be positive. Transcripts will be coded line by line.

Synthesis with intervention uptake data

We will synthesise key aspects of process evaluation data, with effectiveness of the NAT-C within clusters according to randomisation strata, to improve understanding using NPT about how and if the NAT-C should be implemented into clinical practice using Critical Interpretative Synthesis (CIS).[39]

Kirkpatrick's model for training evaluation will be used to evaluate NAT-C training in terms of: reaction to the training, learning and skills improvement, behavioural change and results.[40] Reaction will be assessed by responses to NoMAD surveys and interview. Learning and behavioural change will be evaluated through qualitative data.

Trial organization and governance

CANAssess is sponsored by the University of Hull (UoH) coordinated by Leeds CTRU and UoH. The sponsor had no direct input in to the design or conduct of the study. The Trial Management Group consists (TMG) of co-applicants, trial coordinators, four GP-hub leads and a public-patient representative. The TMG is responsible for clinical set-up, on-going management, promotion of the trial, and for the interpretation and publishing of the results. A TSC will meet annually and on request to provide independent oversight of the trial and reports to the Sponsor.

A Data Monitoring and Ethics Committee (DMEC) is not needed due to the nature of the study. The TSC will adopt a safety monitoring role, with the constitution of a sub-committee to review safety issues where necessary.

Patient and public involvement

An experienced lay representative was part of our funding application. She also reviewed and edited public-facing study documentation, and sits on our TMG, with public-patient involvement as a standing item. A further lay representative forms part of our TSC.

Ethics and Dissemination

For peer review only - http://bmjopen.bmj.com/site/about/guidelines.xhtml

Dissemination

If trial results are positive, the NAT-C has the potential to become the gold standard cancer care delivery in primary care as the only valid tool subjected to formal effectiveness testing.

Findings will be presented and discussed at a final dissemination meeting, to which a wide range of stakeholders will be invited, including trial clinicians, participants and those involved in the stakeholder engagement.

Results of the study will be published in peer-review publications and will be presented at national and international conferences. A lay summary of our findings will be published on study and organizational websites and will be accessible to participants.

Ethical considerations

The trial received ethical approval from the London-Surrey REC (20/LO/0312). Any future amendments to the trial will be submitted to the REC and participants will be informed of any changes which may affect them.

For peer review only

For peer review only - http://bmjopen.bmj.com/site/about/guidelines.xhtml

Impact of COVID-19

The COVID-19 outbreak in England occurred just as ethical approval for the study had been obtained and the process of site identification had begun. We halted site identification and adapted the trial processes to allow remote intervention delivery as per practice procedure for remote consultations, telephone consent and data collection, and online patient study responses and online completion of follow-up questionnaires. Amidst concerns that patient recruitment may be affected by social distancing measures, the Leeds CTRU also highlighted how their secure online computer systems would allow online informed consent provision and data collection. We therefore submitted an amendment to allow all study activity to be completed remotely through phone or video-conference.

Trial status

Following COVID-related delays, the trial team is in place, incorporating employed trial-specific research nurses and Clinical Research Network (CRN) support. Recruitment of GP practices and participants is underway. Our first study site was opened for recruitment on 21.10.2020 and we now seven general practices recruiting participants. The first participant was recruited on 01/12/2020 and as of 16.03.2021 we have recruited 36 participants. This manuscript has been prepared in accordance with study protocol v.3, 24.06.2020. A copy of the full protocol is available on request from Dr Joseph Clark.

Trial registration

ISRCTN15497400, registered 07/04/2020

Funding

This work was supported by Yorkshire Cancer Research, grant number: H423

Authors' contributions

All authors meet the International Committee of Medical Journal Editors (ICMJE) criteria for authorship and contributed to the work presented as follows: conception and design of the trial (JC, AW-H, DM, RF, SW, JD, AF, PB, EM, MJ), development of data analysis methods (AF, BC, DM, AW-H), process evaluation methods (JC/MJ). JC produced a first draft the manuscript, after which all authors commented and provided edits ahead of finalisation.

Correspondence should be directed to: Dr Joseph Clark, e: joseph.clark@hyms.ac.uk

Competing interests

All authors declare no competing interests.

References

[1] Ruijs, C.D., Kerkhof, A.J., van der Wal, G. et al. Symptoms, unbearability and the nature of suffering in terminal cancer patients dying at home: a prospective primary care study. *BMC Fam Pract* 14, 201 (2013). https://doi.org/10.1186/1471-2296-14-201

[2] Adams E, Boulton M and Rose P et al. Views of cancer care reviews in primary care: a qualitative study. *BJGP,* 2011;61(585):e173-e183.

[3] Waller A, Girgis A, Johnson C, et al. Improving outcomes for people with progressive cancer: interrupted time series trial of a needs assessment intervention. *J Pain Symptom Manage* 2012;43:569–81.

For peer review only

For peer review only - http://bmjopen.bmj.com/site/about/guidelines.xhtml

[4] Homsi J, Walsh D, Rivera N, Rybicki LA, Nelson KA, Legrand SB, Davis M, Naughton M, Gvozdjan D and Pham H. Symptom evaluation in palliative medicine: patient report vs systematic assessment, *Support Care Cancer,* 2006;14(5):444-53.
[5] Carlson LE, Waller A, Mitchell AJ. Screening for distress and unmet needs in patients with cancer: review and recommendations. J Clin Oncol. 2012;30(11):1160-77.
[6] Department of Health and Social Care. Living With and Beyond Cancer: Taking Action to Improve Outcomes 2013 [Available from: https://assets.publishing.service.gov.uk/government/uploads/system/uploads/attachment_data/file/181054/9333-TSO-2900664-NCSI_Report_FINAL.pdf.
[7] Foy R, Leaman B, McCrorie C, Petty D, House A, Bennett M, Carder P, Faulkner S, Glidewell L, West R. Prescribed opioids in primary care: cross sectional and longitudinal analyses of influence of patient and practice characteristics. BMJ Open 2016;6:e010276. doi:10.1136/bmjopen-2015-010276.
[8] Tamburini M, Gangeri L, Brunelli C et al. Assessment of hospitalised cancer patients' needs by the Needs Evaluation Questionnaire. *Ann Oncol,* 2000; 11(1):31-37.
[9] Davidson PM, Paull G, Introna K et al. Integrated, collaborative palliative care in heart failure: the St. George Heart Failure Service experience 1999-2002*. J Cardiovasc Nurs,* 2004; 19(1):68-75.
[10] Deeken JF, Taylor KL, Mangan P, Yabroff KR, Ingham JM. Care for the caregivers: a review of self-report instruments developed to measure the burden, needs, and quality of life of informal caregivers. *J Pain Symptom Manage,* 2003; 26(4):922-953.
[11] Wen KY, Gustafson DH. Needs assessment for cancer patients and their families. *Health Qual Life Outcomes,* 2004; 2:11.
[12] Girgis A, Johnson C, Currow DC, et al. Palliative Care Needs Assessment Guidelines. The Centre for Health Research & Psycho-oncology. 2006. Newcastle, NSW.
[13] Girgis A, Johnson C, Currow D, Waller A, Kristjanson L, Mitchell G, et al. Palliative care needs assessment guidelines. 2006
[14] Groenvold M, Petersen MA, Aaronson NK, Arraras JI, Blazeby JM, Bottomley A, et al. EORTC QLQ-C15-PAL: the new standard in the assessment of health-related quality of life in advanced cancer? Palliat Med. 2006;20(2):59-61.
[15] Groenvold M, Petersen MA, Aaronson NK, Arraras JI, Blazeby JM, Bottomley A, et al. The development of the EORTC QLQ-C15-PAL: a shortened questionnaire for cancer patients in palliative care. Eur J Cancer. 2006;42(1):55-64.
[16] Osse BHP, Vernooij-Dassen MJFJ, de Vree BPW, Schade E, Grol RPTM. Assessment of the need for palliative care as perceived by individual cancer patients and their families - A review of instruments for improving patient participation in palliative care. Cancer. 2000;88(4):900-11.
[17] Allgar VL, Chen H, Richfield E, Currow D, Macleod U, Johnson MJ. Psychometric Properties of the Needs Assessment Tool-Progressive Disease Cancer in U.K. Primary Care. *J Pain Symptom Manage*, 2018 Oct;56(4):602-612. doi: 10.1016/j.jpainsymman.2018.07.002. Epub 2018 Aug 7. PMID: 30009964.
[18] Clark J, Amoakwa E and Wright-Hughes A et al. A cluster randomised trial of a Needs Assessment Tool for adult Cancer patients and their carers (NAT-C) in primary care: A feasibility study. *Plos One,* 2021; https://doi.org/10.1371/journal.pone.0245647.
[19] Boyes A, Girgis A, Lecathelinais C. Brief assessment of adult cancer patients' perceived needs: development and validation of the 34-item Supportive Care Needs Survey (SCNS-SF34). J Eval Clin Pract. 2009;15(4):602-6.
[20] Abernethy AP, Shelby-James T, Fazekas BS, Woods D, Currow DC. The Australia-modified Karnofsky Performance Status (AKPS) scale: a revised scale for contemporary palliative care clinical practice [ISRCTN81117481]. BMC Palliat Care. 2005;4:7.
[21] Services AH. Guidelines for using the revised Edmonton Symptom Assessment System (ESAS-r) [Website]. 2010 [Available from: http://www.palliative.org/NewPC/_pdfs/tools/3C7%20ESAS-r%20guidelines%20Aug%2022%202014.pdf.
[22] Groenvold M, Petersen MA, Aaronson NK, Arraras JI, Blazeby JM, Bottomley A, et al. EORTC QLQ-C15-PAL: the new standard in the assessment of health-related quality of life in advanced cancer? Palliat Med. 2006;20(2):59-61.
[23] Goranitis I, Coast J, Al-Janabi H. An investigation into the construct validity of the Carer Experience Scale (CES). Qual Life Res. 2014;23(6):1743-52.

For peer review only - http://bmjopen.bmj.com/site/about/guidelines.xhtml

[24] Higginson IJ, Gao W, Jackson D, Murray J, Harding R. Short-form Zarit Caregiver Burden Interviews were valid in advanced conditions. J Clin Epidemiol. 2010;63(5):535-42
[25] Herdman M, Gudex C, Lloyd A, Janssen M, Kind P, Parkin D, et al. Development and preliminary testing of the new five-level version of EQ-5D (EQ-5D-5L). Qual Life Res. 2011;20(10):1727-36.
[26] Huynh E, Coast J, Rose J, Kinghorn P and Flynn T. Values for the ICECAP-Supportive Care Measure (ICECAP-SCM) for use in economic evaluation at end of life. *Soc Sci Med,* 2017;189:114-128.
[27] NHS Digital. Organisation Data Service 2019 [Available from: https://digital.nhs.uk/services/organisation-data-service.
[28] NHS Digital. NHS Payments to General Practice - England, 2018/19 2019 [Available from: https://digital.nhs.uk/data-and-information/publications/statistical/nhs-payments-to-general-practice/england-2018-19. Accessed 30.10.2020.
[29] NHS England. Standard General Medical Services Contract 2018/19 2019 [Available from: https://www.england.nhs.uk/wp-content/uploads/2019/04/general-medical-services-contract-19-20.pdf.
[30] McElduff P, Boyes A, Zucca A, Girgis AJNCfHR, Psycho-oncology. Supportive Care Needs Survey: A guide to administration, scoring and analysis. Newcastle: Centre for Health Research and Psycho-oncology; 2004.
[31] Campbell MK, Fayers PM, Grimshaw JM. Determinants of the intracluster correlation coefficient in cluster randomized trials: the case of implementation research. Clin Trials. 2005;2(2):99-107.
[32] Bolzern JE, Mitchell A, Torgerson DJ. Baseline testing in cluster randomised controlled trials: should this be done? BMC Med Res Methodol. 2019;19(1):106.
[33]Higginson IJ, Evans CJ, Grande G, et al. . Evaluating complex interventions in end of life care: the MORECare statement on good practice generated by a synthesis of transparent expert consultations and systematic reviews. BMC Med 2013;11:11 10.1186/1741-7015-11-111
[34] National Institute for Health and Clinical Excellence (NICE). Guide to the methods of technology appraisal London: NICE; 2013 [Available from: https://www.nice.org.uk/process/pmg9/resources/guide-to-the-methods-of-technology-appraisal-2013-pdf-2007975843781.
[35] Barton GR, Briggs AH, Fenwick EA. Optimal cost-effectiveness decisions: the role of the cost-effectiveness acceptability curve (CEAC), the cost-effectiveness acceptability frontier (CEAF), and the expected value of perfection information (EVPI). Value in health. 2008 Sep;11(5):886-97.
[36] Willan AR, Briggs AH, Hoch JS. Regression methods for covariate adjustment and subgroup analysis for non-censored cost-effectiveness data. Health economics. 2004 May;13(5):461-75.
[37] Silverman D. Doing Qualitative Research. 3rd Edition ed. London: Sage; 2009.
[38] Normalization Process Theory. Core Propositions of NPT 2019 [Available from: http://www.normalizationprocess.org/theory-behind-npt/core-propositions-of-npt/.
[39] Dixon-Woods M, Cavers D and Agarwal S et al. Conducting a critical interpretive synthesis of the literature on access to healthcare by vulnerable groups. *BMC Med Res Method,* 2006;6@53. doi:10.1186/1471-2288-6-35.
[40] Barr H, Freeth D, Hammick M, Koppel I, Reeves S. Evaluations of interprofessional education: a United Kingdom review for health and social care. 2000. Available from: https://www.caipe.org/resources/publications/barr-h-freethd-hammick-m-koppel-i-reeves-s-2000-evaluations-of-interprofessional-education. 30.10.2020.

For peer review only - http://bmjopen.bmj.com/site/about/guidelines.xhtml

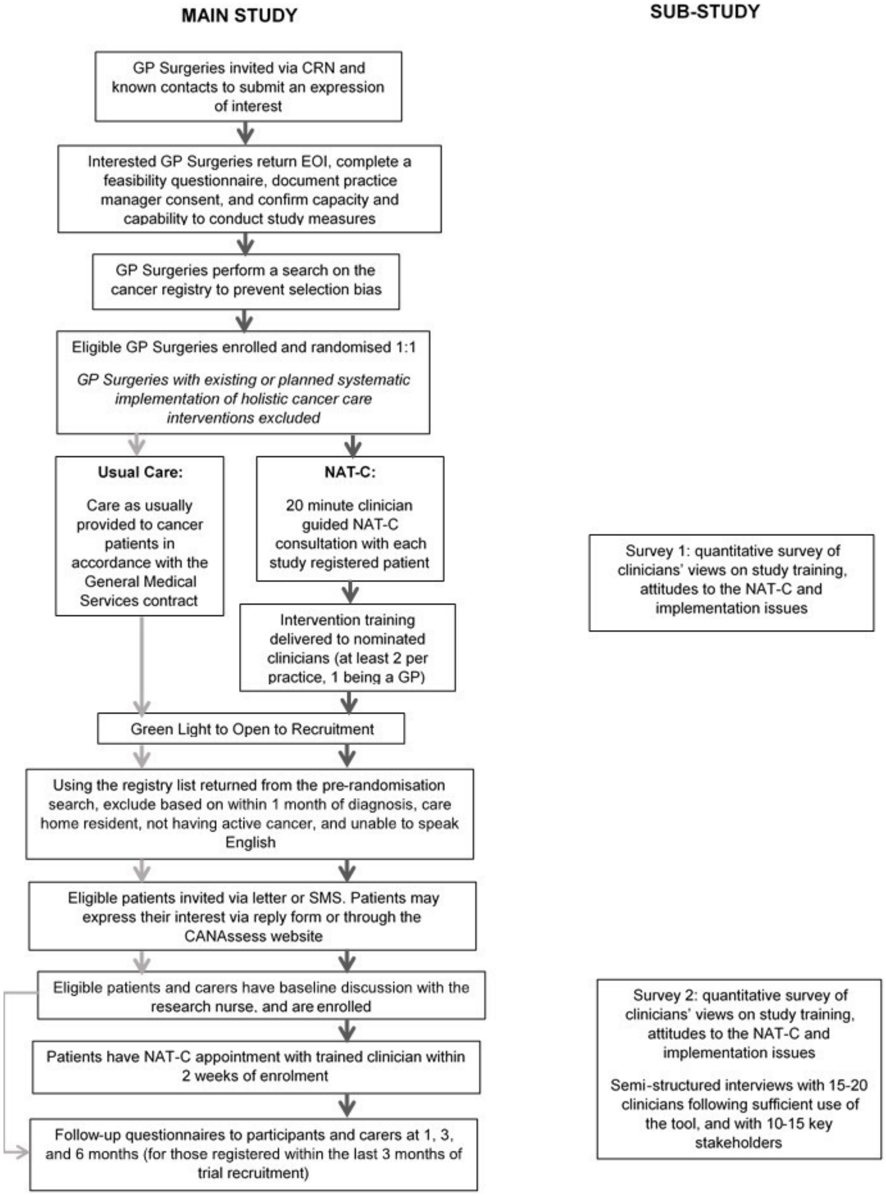

Figure 1: Study Flow Chart

58x79mm (300 x 300 DPI)

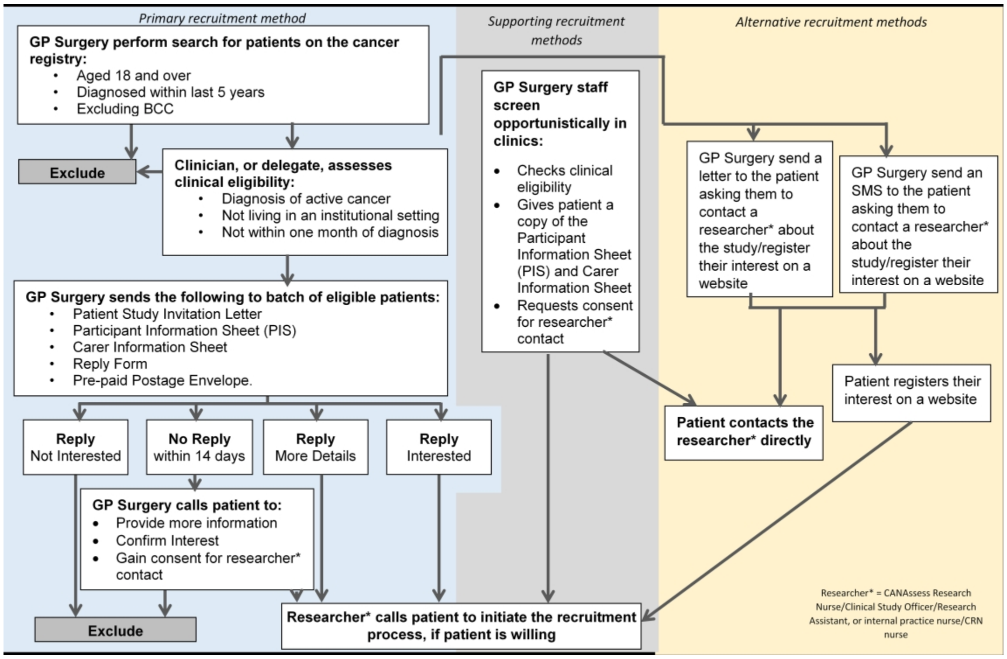

Figure 2. Participant and carer recruitment

536x349mm (300 x 300 DPI)

Supplementary file 1 –Risk of bias assessment

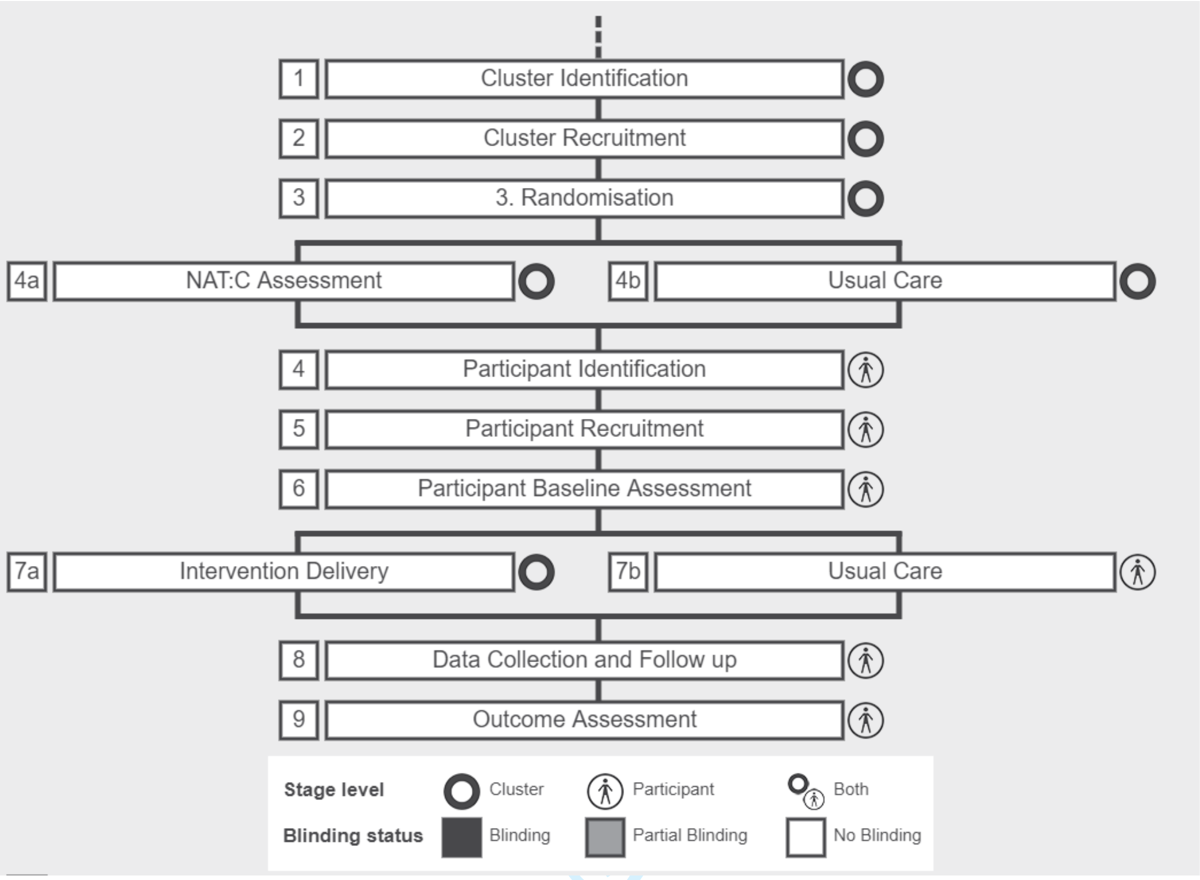

1. Cluster identification

General Practices in Yorkshire and Tyne and Wear will be approached and 54 recruited. Cluster identification will be conducted by four separate 'hubs', located at Leeds, Hull, Sheffield and the North East of England, each co-ordinated by a clinical 'hub-lead'. Practices will be invited to submit an Expression of Interest through relevant Clinical Research Network (CRN) mailing lists and hub lead networks. Practices will be asked to confirm their capacity to deliver the trial and eligibility will be assessed by the research team in terms of local research capacity.

2. Cluster recruitment

General Practices will be eligible unless they: took part in the feasibility study, have implemented or are planning to implement within the duration of the trial a systematic holistic cancer care intervention that overlaps with the NAT-C, or are unable to confirm capacity and capability to deliver the study at their GP Surgery. Practices will provide consent to deliver the study on the terms stated in a Schedule of Events Cost Attribution Template (SoECAT).

3. Randomisation

General practices will be randomised with a 1:1 ratio level by a statistician at the Leeds Clinical Trials Unit. Randomisation will take place post-site initiation. Practices will be randomised to either i) Needs Assessment Tool – Cancer NAT-C) plus Usual Care or ii) Usual Care, stratified by: Locality; Urban or rural area (UK government rural-urban classification based on GP Surgery postcode; List Size: <5000, 5000-10000, >10000 (obtained from NHS digital); A GP training practice (obtained from

For peer review only - http://bmjopen.bmj.com/site/about/guidelines.xhtml

site feasibility questionnaire): Yes, No. Practices will be randomised after consent and prior to study training and participant identification. Training will take place either face to face, via video-link or with a piloted online training package.

4. Participant Identification

An administrator or research nurse will conduct a database search for patients with 'active cancer' post-randomisation. A date restriction of five years will be applied in terms of date of diagnosis. This will remove historic cancer cases from the results, but may miss patients who have been living with active cancer for more than five years. A further exclusion will remove people with basal cell carcinoma (BCC) using a read code. A clinician will assess participant eligibility, in particular, to confirm a current cancer diagnosis and to confirm capacity to provide informed consent. There is a small risk that clinicians may exclude patients due to stage of illness. The research team will encourage clinicians to give patients at any stage of illness the opportunity to take part. Eligibility will be defined by a clinic and eligibility checks will take place during study monitoring conducted by a trained researcher.

5. Participant Recruitment

Eligible patients will be invited to the study either via letter, SMS or opportunistically at General Practices. All eligible patients will be provided with a Study Invitation Sheet. A Research Nurse will contact patients expressing interest in the study, answer any questions that the patient may and arrange informed written consent. Witnessed informed consent may be taken if a patient is unable to write.

Participating patients will be given the opportunity to nominate a carer if they would like to. Nominated carers will then receive a Carer Information Sheet and a Study Invitation. A Research Nurse will answer any questions that carer may have about the study ahead of arranging informed written consent.

Participants will find out which arm of the trial their practice has been allocated to after providing informed consent.

6. Participant Baseline Assessment

After taking written informed consent, a Research Nurse will help participants to provide baseline information. Demographic information and clinical characteristics will be collected. During a face to face appointment, a research nurse will collect participant: age, sex, cancer type and stage, treatment history, ethnicity, relationship status, living arrangement and accommodation, household income, postcode, the Australian Karnofsky Performance Status (AKPS) and the Charlson Co-morbidity Index. Patients will then be advised regarding their allocation and advised how to proceed win the study.

7a. Intervention Delivery

General Practitioners and clinical nurses will receive training in how to use the NAT:C either face to face or online. Research nurses will not receive intervention training. General practices will then contact patients to arrange a twenty minute needs assessment appointment, to occur within two weeks of informed consent. Clinicians will conduct a twenty-minute needs assessment appointment using the NAT-C. The NAT-C will be available as a template on EMIS and SystmOne and a paper copy will be available to clinicians if required. Patients may attend their needs assessment appointment

For peer review only

with a carer if they would like to. The carer does not have to be participating in the study. Patients will also have access their General Practice as usual.

7b. Usual Care

Patients will have access to their General Practice as usual.

8. Data collection and follow up

Patient participants will be asked to complete follow up questionnaires at one month and three months: the Supportive Care Needs Survey, the AKPS, the revised Edmonton Symptom Assessment System (ESAS-r), the EORTC QLQ-C15-PAL and a bespoke Resource Use Questionnaire (RUQ). Participants will be supported by a research nurse during data collection either face to face or over the phone. Completed NAT:C assessments will be retrieved from the practice clinical record. It will not be possible to blind research nurses to the allocation of General Practices (and therefore patients) during data collection. However, data collection will not be conducted by anybody who has been involved in delivering the intervention. Data collection will be undertaken as close to the stated time points as feasible.

9. Outcome assessment

Primary outcome will be proportion of patients with an unmet need on the SCNS. Analysis will be conducted on an intent to treat basis. Final analysis will be conducted by a senior statistician at the Leeds Clinical Trials Unit and will take place once all participants have completed three month measures.

For peer review only

TIDieR Template for Intervention Description and Replication

The TIDieR (Template for Intervention Description and Replication) Checklist*:

Information to include when describing an intervention and the location of the information

Item number	Item	Where located ** Primary paper (page or appendix number)	Other † (details)
	**BRIEF NAME**		
**1.**	Provide the name or a phrase that describes the intervention.	______4,5______	______________
	**WHY**		
**2.**	Describe any rationale, theory, or goal of the elements essential to the intervention.	________4,5,9____	______________
	**WHAT**		
**3.**	Materials: Describe any physical or informational materials used in the intervention, including those provided to participants or used in intervention delivery or in training of intervention providers. Provide information on where the materials can be accessed (e.g. online appendix, URL).	_______9_____	______________
**4.**	Procedures: Describe each of the procedures, activities, and/or processes used in the intervention, including any enabling or support activities.	_________9___	______________
	**WHO PROVIDED**		
**5.**	For each category of intervention provider (e.g. psychologist, nursing assistant), describe their expertise, background and any specific training given.	_______9_____	______________
	**HOW**		
**6.**	Describe the modes of delivery (e.g. face-to-face or by some other mechanism, such as internet or telephone) of the intervention and whether it was provided individually or in a group.	_______9_____	______________
	**WHERE**		

For peer review only

For peer review only - http://bmjopen.bmj.com/site/about/guidelines.xhtml

**7.**	Describe the type(s) of location(s) where the intervention occurred, including any necessary infrastructure or relevant features.	________9____ _	______________
	**WHEN and HOW MUCH**		
**8.**	Describe the number of times the intervention was delivered and over what period of time including the number of sessions, their schedule, and their duration, intensity or dose.	__________9___ _	______________
	**TAILORING**		
**9.**	If the intervention was planned to be personalised, titrated or adapted, then describe what, why, when, and how.	___________9__ _	______________
	**MODIFICATIONS**		
**10.‡**	If the intervention was modified during the course of the study, describe the changes (what, why, when, and how).	______NA____ ___	______________
	**HOW WELL**		
**11.**	Planned: If intervention adherence or fidelity was assessed, describe how and by whom, and if any strategies were used to maintain or improve fidelity, describe them.	______________	______________
**12.‡**	Actual: If intervention adherence or fidelity was assessed, describe the extent to which the intervention was delivered as planned.	__________NA_ ___	______________

** **Authors** - use N/A if an item is not applicable for the intervention being described. **Reviewers** – use '?' if information about the element is not reported/not sufficiently reported.

† If the information is not provided in the primary paper, give details of where this information is available. This may include locations such as a published protocol or other published papers (provide citation details) or a website (provide the URL).

‡ If completing the TIDieR checklist for a protocol, these items are not relevant to the protocol and cannot be described until the study is complete.

* We strongly recommend using this checklist in conjunction with the TIDieR guide (see *BMJ* 2014;348:g1687) which contains an explanation and elaboration for each item.

* The focus of TIDieR is on reporting details of the intervention elements (and where relevant, comparison elements) of a study. Other elements and methodological features of studies are covered by other reporting statements and checklists and have not been duplicated as part of the TIDieR checklist. When a **randomised trial** is being reported, the TIDieR checklist should be used in conjunction with the CONSORT statement (see www.consort-statement.org) as an extension of **Item 5 of the CONSORT 2010 Statement.** When a **clinical trial protocol** is being reported, the TIDieR checklist should be used in conjunction with the SPIRIT statement as an extension of **Item 11 of the SPIRIT 2013**

For peer review only - http://bmjopen.bmj.com/site/about/guidelines.xhtml
For peer review only

Statement (see www.spirit-statement.org). For alternate study designs, TIDieR can be used in conjunction with the appropriate checklist for that study design (see www.equator-network.org).

For peer review only

For peer review only - http://bmjopen.bmj.com/site/about/guidelines.xhtml

SPIRIT
STANDARD PROTOCOL ITEMS: RECOMMENDATIONS FOR INTERVENTIONAL TRIALS

SPIRIT 2013 Checklist: Recommended items to address in a clinical trial protocol and related documents*

Section/item	Item No	Description	Page Number on which item is reported
**Administrative information**			
Title	1	Descriptive title identifying the study design, population, interventions, and, if applicable, trial acronym	3
Trial registration	2a	Trial identifier and registry name. If not yet registered, name of intended registry	16
	2b	All items from the World Health Organization Trial Registration Data Set	3,4, 7, 10, 14, 16
Protocol version	3	Date and version identifier	16
Funding	4	Sources and types of financial, material, and other support	16
Roles and responsibilities	5a	Names, affiliations, and roles of protocol contributors	16
	5b	Name and contact information for the trial sponsor	16
	5c	Role of study sponsor and funders, if any, in study design; collection, management, analysis, and interpretation of data; writing of the report; and the decision to submit the report for publication, including whether they will have ultimate authority over any of these activities	16
	5d	Composition, roles, and responsibilities of the coordinating centre, steering committee, endpoint adjudication committee, data management team, and other individuals or groups overseeing the trial, if applicable (see Item 21a for data monitoring committee)	16
**Introduction**			

For peer review only

Background and rationale	6a	Description of research question and justification for undertaking the trial, including summary of relevant studies (published and unpublished) examining benefits and harms for each intervention	3-4
	6b	Explanation for choice of comparators	9
Objectives	7	Specific objectives or hypotheses	4-5
Trial design	8	Description of trial design including type of trial (eg, parallel group, crossover, factorial, single group), allocation ratio, and framework (eg, superiority, equivalence, noninferiority, exploratory)	4
**Methods: Participants, interventions, and outcomes**			
Study setting	9	Description of study settings (eg, community clinic, academic hospital) and list of countries where data will be collected. Reference to where list of study sites can be obtained	5
Eligibility criteria	10	Inclusion and exclusion criteria for participants. If applicable, eligibility criteria for study centres and individuals who will perform the interventions (eg, surgeons, psychotherapists)	5, 6, 13, 14
Interventions	11a	Interventions for each group with sufficient detail to allow replication, including how and when they will be administered	9,10
	11b	Criteria for discontinuing or modifying allocated interventions for a given trial participant (eg, drug dose change in response to harms, participant request, or improving/worsening disease)	NA
	11c	Strategies to improve adherence to intervention protocols, and any procedures for monitoring adherence (eg, drug tablet return, laboratory tests)	4,5
	11d	Relevant concomitant care and interventions that are permitted or prohibited during the trial	9
Outcomes	12	Primary, secondary, and other outcomes, including the specific measurement variable (eg, systolic blood pressure), analysis metric (eg, change from baseline, final value, time to event), method of aggregation (eg, median, proportion), and time point for each outcome. Explanation of the clinical relevance of chosen efficacy and harm outcomes is strongly recommended	4,5

For peer review only - http://bmjopen.bmj.com/site/about/guidelines.xhtml

Participant timeline	13	Time schedule of enrolment, interventions (including any run-ins and washouts), assessments, and visits for participants. A schematic diagram is highly recommended (see Figure)	9,10
Sample size	14	Estimated number of participants needed to achieve study objectives and how it was determined, including clinical and statistical assumptions supporting any sample size calculations	11
Recruitment	15	Strategies for achieving adequate participant enrolment to reach target sample size	6,7
**Methods: Assignment of interventions (for controlled trials)**			
Allocation:			
Sequence generation	16a	Method of generating the allocation sequence (eg, computer-generated random numbers), and list of any factors for stratification. To reduce predictability of a random sequence, details of any planned restriction (eg, blocking) should be provided in a separate document that is unavailable to those who enrol participants or assign interventions	6
Allocation concealment mechanism	16b	Mechanism of implementing the allocation sequence (eg, central telephone; sequentially numbered, opaque, sealed envelopes), describing any steps to conceal the sequence until interventions are assigned	6
Implementation	16c	Who will generate the allocation sequence, who will enrol participants, and who will assign participants to interventions	6
Blinding (masking)	17a	Who will be blinded after assignment to interventions (eg, trial participants, care providers, outcome assessors, data analysts), and how	NA
	17b	If blinded, circumstances under which unblinding is permissible, and procedure for revealing a participant's allocated intervention during the trial	NA
**Methods: Data collection, management, and analysis**			

Data collection methods	18a	Plans for assessment and collection of outcome, baseline, and other trial data, including any related processes to promote data quality (eg, duplicate measurements, training of assessors) and a description of study instruments (eg, questionnaires, laboratory tests) along with their reliability and validity, if known. Reference to where data collection forms can be found, if not in the protocol	9,10
	18b	Plans to promote participant retention and complete follow-up, including list of any outcome data to be collected for participants who discontinue or deviate from intervention protocols	9, 10
Data management	19	Plans for data entry, coding, security, and storage, including any related processes to promote data quality (eg, double data entry; range checks for data values). Reference to where details of data management procedures can be found, if not in the protocol	7
Statistical methods	20a	Statistical methods for analysing primary and secondary outcomes. Reference to where other details of the statistical analysis plan can be found, if not in the protocol	11,12,
	20b	Methods for any additional analyses (eg, subgroup and adjusted analyses)	12,13,14
	20c	Definition of analysis population relating to protocol non-adherence (eg, as randomised analysis), and any statistical methods to handle missing data (eg, multiple imputation)	12
**Methods: Monitoring**			
Data monitoring	21a	Composition of data monitoring committee (DMC); summary of its role and reporting structure; statement of whether it is independent from the sponsor and competing interests; and reference to where further details about its charter can be found, if not in the protocol. Alternatively, an explanation of why a DMC is not needed	15
	21b	Description of any interim analyses and stopping guidelines, including who will have access to these interim results and make the final decision to terminate the trial	11,12

Harms	22	Plans for collecting, assessing, reporting, and managing solicited and spontaneously reported adverse events and other unintended effects of trial interventions or trial conduct	11
Auditing	23	Frequency and procedures for auditing trial conduct, if any, and whether the process will be independent from investigators and the sponsor	11
**Ethics and dissemination**			
Research ethics approval	24	Plans for seeking research ethics committee/institutional review board (REC/IRB) approval	15
Protocol amendments	25	Plans for communicating important protocol modifications (eg, changes to eligibility criteria, outcomes, analyses) to relevant parties (eg, investigators, REC/IRBs, trial participants, trial registries, journals, regulators)	15
Consent or assent	26a	Who will obtain informed consent or assent from potential trial participants or authorised surrogates, and how (see Item 32)	7
	26b	Additional consent provisions for collection and use of participant data and biological specimens in ancillary studies, if applicable	NA
Confidentiality	27	How personal information about potential and enrolled participants will be collected, shared, and maintained in order to protect confidentiality before, during, and after the trial	10
Declaration of interests	28	Financial and other competing interests for principal investigators for the overall trial and each study site	16
Access to data	29	Statement of who will have access to the final trial dataset, and disclosure of contractual agreements that limit such access for investigators	10
Ancillary and post-trial care	30	Provisions, if any, for ancillary and post-trial care, and for compensation to those who suffer harm from trial participation	NA
Dissemination policy	31a	Plans for investigators and sponsor to communicate trial results to participants, healthcare professionals, the public, and other relevant groups (eg, via publication, reporting in results databases, or other data sharing arrangements), including any publication restrictions	15

For peer review only

	31b	Authorship eligibility guidelines and any intended use of professional writers	16
	31c	Plans, if any, for granting public access to the full protocol, participant-level dataset, and statistical code	16
**Appendices**			
Informed consent materials	32	Model consent form and other related documentation given to participants and authorised surrogates	NA
Biological specimens	33	Plans for collection, laboratory evaluation, and storage of biological specimens for genetic or molecular analysis in the current trial and for future use in ancillary studies, if applicable	NA

*It is strongly recommended that this checklist be read in conjunction with the SPIRIT 2013 Explanation & Elaboration for important clarification on the items. Amendments to the protocol should be tracked and dated. The SPIRIT checklist is copyrighted by the SPIRIT Group under the Creative Commons "Attribution-NonCommercial-NoDerivs 3.0 Unported" license.

For peer review only

BMJ Open

CANcer Patients' Needs Assessment in Primary Care: Study Protocol for a Cluster Randomised Controlled Trial (cRCT), economic evaluation and Normalisation Process Theory evaluation of the Needs Assessment Tool Cancer (CANAssess)

Journal:	*BMJ Open*
Manuscript ID	bmjopen-2021-051394.R1
Article Type:	Protocol
Date Submitted by the Author:	26-Jan-2022
Complete List of Authors:	Clark, Joseph; University of Hull, Wolfson Palliative Care Research Centre<br>Copsey, Bethan; University of Leeds Clinical Trials Research Unit,<br>Wright-Hughes, Alexandra; University of Leeds, Clinical Trials Research Unit<br>McNaught, Emma; University of Leeds, Clinical Trials Research Unit<br>Bijsterveld, Petra; University of Leeds, Clinical Trials Research Unit<br>McCormack, Terry; University of Hull, Hull York Medical School<br>Foy, Robbie; University of Leeds, Leeds Institute of Health Sciences<br>Wilkes, Scott; University of Sunderland, School of Pharmacy<br>Dickson, Jon; The University of Sheffield, Academic Unit of Primary Medical Care<br>Meads, David; University of Leeds, Leeds Institute of Health Sciences<br>Farrin, Amanda; University of Leeds, Clinical Trials Research Unit<br>Johnson, Miriam; The University of Hull, Wolfson Palliative Care Research Centre
**Primary Subject Heading**:	General practice / Family practice
Secondary Subject Heading:	Palliative care
Keywords:	PALLIATIVE CARE, PRIMARY CARE, Adult palliative care < PALLIATIVE CARE

SCHOLARONE™ Manuscripts

Title page

CANcer Patients' Needs Assessment in Primary Care: Study Protocol for a Cluster Randomised Controlled Trial (cRCT), economic evaluation and Normalisation Process Theory evaluation of the Needs Assessment Tool Cancer (CANAssess)

Authors

Dr Joseph Clark, Wolfson Palliative Care Research Centre, University of Hull, United Kingdom

Dr Bethan Copsey, Leeds Institute of Clinical Trials Research, University of Leeds, United Kingdom

Dr Alexandra Wright-Hughes, Leeds Institute of Clinical Trials Research, University of Leeds, United Kingdom

Ms Emma McNaught, Leeds Institute of Clinical Trials Research, University of Leeds, United Kingdom

Ms Petra Bijsterveld, Leeds Institute of Clinical Trials Research, University of Leeds, United Kingdom

Dr Terry McCormack, Hull York Medical School, University of Hull, United Kingdom

Professor Robbie Foy, University of Leeds, United Kingdom

Professor Scott Wilkes, University of Sunderland, United Kingdom

Dr Jon Dickson, University of Sheffield, United Kingdom

Dr David Meads, University of Leeds, United Kingdom

Professor Amanda Farrin, Leeds Institute of Clinical Trials Research, University of Leeds, United Kingdom

Professor Miriam Johnson, Wolfson Palliative Care Research Centre, University of Hull, United Kingdom

Corresponding author

Dr Joseph Clark, Wolfson Palliative Care Research Centre, University of Hull, Cottingham Road, Hull, United Kingdom, HU6 7RX

E: joseph.clark@hyms.ac.uk

T: 01482 347538

F: N/A

Key words

Palliative Care, Primary Health Care, Cancer Care, Cluster Randomised Trial, Economic Evaluation, Normalisation Process Theory

Word count

3954

For peer review only - http://bmjopen.bmj.com/site/about/guidelines.xhtml

Abstract

Introduction: Unmet needs in patients with cancer and their carers are common but poorly identified and addressed. The Needs Assessment Tool-Cancer (NAT-C) is a structured consultation guide to identify and triage patient and carer unmet needs. The NAT-C is validated, but its effectiveness in reducing unmet patient and carer needs in primary care is unknown.

Methods and analysis: Cluster randomised controlled trial with internal pilot and embedded process evaluation to test the clinical and cost effectiveness of the NAT-C in primary care for people with active cancer in reducing unmet patient and carer need, compared with usual care. We will recruit 1080 patients with active cancer (and carers if relevant) from 54 general practices in England.

Participating practices will be randomised 1:1 to either deliver a NAT-guided clinical consultation plus usual care or to usual care alone. Consenting participants with active cancer and their carers (if nominated) will be asked to complete study questionnaires at baseline, one and three months for all, six months except for those recruited outside of the last three months of recruitment, and attend a NAT-C appointment if allocated to an intervention practice. An internal pilot will assess: site and participant recruitment, intervention uptake, and follow-up rates. The primary outcome, the proportion of patients with an unmet need on the Supportive Care Needs Survey Short Form 34 (SCNS-SF34) at three months post registration, will be analysed using a multi-level logistic regression. Mixed-methods process evaluation informed by Normalisation Process Theory will use quantitative survey and interview data from clinicians and key stakeholders in cancer care to develop an implementation strategy for nationwide rollout of the NAT-C if the intervention is cost-effective.

Ethics and dissemination: Ethical approval from London-Surrey REC (20/LO/0312). Results will be peer-reviewed, published and made available to research participants.

Trial registration: ISRCTN15497400, registered 07/04/2020

For peer review only - http://bmjopen.bmj.com/site/about/guidelines.xhtml

Strengths and limitations

- We are testing the clinical and cost effectiveness of the Needs Assessment Tool – Cancer (NAT-C) which has been validated and shown to be acceptable to patients and clinicians during feasibility testing.
- Feasibility testing led to modifications of intervention delivery and informed trial design, making successful completion of the trial more likely.
- CANASSESS is a cluster randomised controlled trial of 54 general practices across two regions in England, making it likely that findings will be generalizable nationwide.
- By necessity, participants, health professionals delivering the intervention and study researchers will be aware of treatment allocation; potential bias will be monitored during the trial.
- COVID-19 presents unique challenges in terms of safely conducting clinical trials in primary care.

For peer review only

For peer review only - http://bmjopen.bmj.com/site/about/guidelines.xhtml

Introduction

Unmet needs in people with cancer and their carers are common but poorly identified and addressed. Many people with cancer experience unmet needs across multiple domains.[1] General Practitioners (GPs, family doctors) and other clinicians in primary care would like to do more to support their cancer patients, but there is no agreed evidence-based best approach.[2] Difficulties are compounded by inconsistent co-ordination of care with oncology services as GPs may be unaware of problems unless patients present directly. However, people with cancer often do not attend primary care for cancer care and systematic, routine holistic assessment of patient problems is rare.[3] In addition, patients commonly volunteer only the most pressing problem to their clinicians; open enquiry in one study only found an average of one problem presented, whereas systematic enquiry discovered an average of ten, many of which were severe and distressing.[4]

Tools are available to assist clinicians caring for people with cancer,[5] but few are designed to identify and triage care needs in the everyday busy clinical setting and across all stages of active disease from diagnosis through to end of life care. Furthermore, although needs assessment tools are advocated,[6] there is no rigorous research evidence to indicate whether they actually improve practice and patient outcomes. A needs assessment tool can reduce unmet needs by providing a consistent and comprehensive approach to prompting discussion patients' range of support and care needs; helps professionals triage tailored action and is useful for audit and service planning.[7,8,9,10,11] Through triage, an assessment tool may help reduce late referrals for palliative care, and improve referrals where there are physical, psychological, social and spiritual problems.[12, 13] However, tools currently available are commonly highly detailed and long for daily clinical use.[14,15,16]

Development of the Needs Assessment Tool Cancer

The Needs Assessment Tool – Cancer (NAT-C) was developed in Australia, where it has been shown to reduce unmet needs of patients in oncology clinics.[3] We adapted and validated this tool for use in UK primary care.[17] Use of the NAT-C aims to reduce unmet supportive and palliative care needs of cancer patients and their carers by supporting systematic clinician assessment of patient and carer needs across multiple domains. Identified problems may be managed in primary care or through referral to other services.

Our Phase II feasibility study found that a randomised trial is feasible in terms of recruitment, data quality, and intervention delivery.[18] Required changes to improve study processes were identified, specifically, confirmation of participant acceptability to be directed to a known NAT-C clinician. Our Resource Use Questionnaire (RUQ) was also modified following feedback from patient participants in the feasibility study. Clinicians, patients and carers also viewed the tool positively and supported need for a definitive trial. A key alteration to the NAT-C was to develop the paper-based tool into digital templates for use in standard electronic clinical record systems (EMIS, SystmOne) in accordance with clinician preferences.

Aims

The CANAssess trial aims to evaluate the clinical effectiveness and cost effectiveness of the NAT-C in reducing unmet needs of patients and carers in primary care carer compared to usual care alone.

Methods and analysis

For peer review only - http://bmjopen.bmj.com/site/about/guidelines.xhtml

Design summary

CANAssess is a multicentre, two-arm, pragmatic, cluster randomised controlled trial (cRCT) with 12-month internal pilot, embedded process evaluation and cost-effectiveness evaluation. A cRCT design reflects that the intervention would be implemented at general practice level and reduces contamination in the control group.

The trial opened to recruitment on 01/10/2020, recruitment is expected to cease on 01/06/2022 and participant follow-up will end 01/09/2022.

Trial objectives and outcomes are reported in Box 1.

Box 1: CANAssess Primary, Secondary, internal pilot, economic and process evaluation objectives

Primary Objective: To test the effectiveness of the NAT-C compared to usual care in reducing unmet patient need as measured using the Supportive Care Needs Survey Short Form 34 (SCNS-SF34[19]) at three months post registration.

Secondary objectives:

To evaluate the effectiveness of the NAT-C compared to usual care with regard to:

- Patient unmet need on psychological, health system information, physical and daily activity, patient care and support, and sexuality domains of the SCNS-SF34 at one, three and six months;
- Patient performance status, measured using the Australian-modified Karnofsky Performance Status (AKPS[20]) at one, three and six months;
- Patient severity of symptoms, measured using the Revised Edmonton Symptom Assessment System (ESAS-r[21]) at one, three and six months;
- Patient mood and quality of life as measured by the European Organisation for Research and Treatment of Cancer Quality of Life-C15-Palliative questionnaire (EORTC QLQ-C15-PAL[22]) at one, three and six months; and
- Carers' ability to care and carer wellbeing as measured using the Carer Experience Scale (CES[23]) and Zarit Burden Interview-12 (ZBI[24]) at one, three and six months.

To evaluate intervention delivery, uptake and fidelity of the NAT-C as measured by:

- NAT-C training of General Practitioners (GPs) and nurses in each general practice;
- Completed NAT-C consultations by patient and general practice (including completion of individual items of the NAT-C);
- Length of NAT-C consultations; and
- Referral patterns and actions taken to meet identified unmet need (including referrals to health professionals and/or services) from the completed NAT-C.

INTERNAL PILOT objectives:
To assess sufficiency of numbers of general practices and patients at 12 months post start of recruitment; we will proceed with the trial unchanged if we have 80% (43) sites open and are recruiting to 80% (48 participants per month) of target. We will assess intervention uptake, follow-up rates, and potential for selection bias.

HEALTH ECONOMIC objectives:
Service utilisation, referral patterns and cost-effectiveness measured using:

For peer review only - http://bmjopen.bmj.com/site/about/guidelines.xhtml

- Bespoke Resource Use Questionnaire (RUQ) for capturing patient healthcare service utilisation and referral patterns at one, three and six months; and
- The EQ-5D-5L[25], ICEpop CAPability Supportive Care Measure (ICECAP-SCM[26]) and CES to generate Quality-Adjusted Life Years (QALYs) and estimates of well-being at one, three and six months.

PROCESS EVALUATION objectives
To assess the adequacy of NAT-C training, intervention fidelity, possible mechanisms of action and issues regarding implementation in practice if the intervention is effective.

Recruitment setting

The study aims to recruit patients and their carers from 54 general practices (clusters) from 4 geographical regions (recruitment "hubs") in Yorkshire, East Midlands and the North East of England. Locations were selected to ensure a range of multi-ethnic, rural and urban populations to maximise generalisability of findings.

Recruitment of general practices

Site identification and recruitment is detailed in Figure 1. General practices will be eligible unless they: took part in the feasibility study, have or are planning to implement within the duration of the trial a systematic holistic cancer care intervention that overlaps with the NAT-C, or lack capacity and capability to deliver the study.

Figure 1: Study Flow Chart

Cluster Randomisation

Where practice manager agreement is obtained, capacity and capability confirmed, and initial read-code search completed, participating general practices (clusters, n=54) will be randomised sequentially via an automated system at the Clinical Trials Research Unit (CTRU). General practice randomisation will be 1:1 to: implement the NAT-C in addition to usual care, or usual care alone, using a computer-generated minimisation programme incorporating a random element to ensure arms are balanced for stratification factors:

- Locality: Urban or rural area.[27, 28]
- List Size: <5000, 5000-10000, >10000[28]
- A GP training practice (obtained from site feasibility questionnaire): Yes, No

General practices and research nurses providing participant recruitment and follow-up support across multiple surgeries will, by necessity, be aware of treatment allocation. However, no member of the research team will be involved with intervention delivery to minimise performance bias. A structured risk of bias assessment is presented in Supplementary File 1. Participating practices will be free to withdraw from the study without negative consequence. In the event of practice withdrawal, we will inquire about reasons for withdrawal and may recruit replacement practices.

Participant eligibility

Eligibility criteria are shown in Box 2

Box 2: Patient/Carer Inclusion/Exclusion

For peer review only - http://bmjopen.bmj.com/site/about/guidelines.xhtml

Patient Inclusion Criteria

- Adults (aged 18 years and above)
- Diagnosis of active cancer (receiving anti-cancer treatment both with curative or palliative intent; managed with "watch and wait"; recurrent or metastatic; or inoperable)
- Willing and able to complete questionnaires at the trial follow-up schedule
- Provision of written or observed verbal informed consent.
- Sufficient knowledge of the English language to provide informed consent and complete trial questionnaires. The use of an appropriate translator/interpreter is allowed.

Patient Exclusion Criteria

- Patients in complete remission (no clinical or radiological evidence of cancer, and at least one month post anti-cancer treatments)
- Patients with basal cell carcinoma
- Patients living in a care home or other institutional setting
- Patients within one month of receiving their initial cancer diagnosis.

Carer Inclusion Criteria

- Adults (aged 18 and above)
- Nominated by participant
- Able to complete trial measures
- Written or observed verbal informed consent.
- Sufficient knowledge of the English language to provide informed consent and complete trial questionnaires. The use of an appropriate translator/interpreter is allowed.

Carer Exclusion Criteria

- Employed to look after the participant.

Participant recruitment

General practices will identify eligible patients by searching cancer registers and screening for eligibility. Eligible patients will be sent a letter with a Patient Information Sheet and expression of interest form. General practices may also send an SMS text message or amended letter to patients inviting them to express interest in the study on the CANAssess website. Patients will provide informed consent (Supplementary File 2) ahead of registration into the study. Consented patients may nominate carers for participation in the trial. Carers agreeing to participate will provide consent. The full process of participant recruitment is presented in Figure 2. For any participant or carer who wishes to withdraw from the trial, we will collect a reason for withdrawal and cease data collection, but keep collected data unless otherwise requested.

Figure 2. Participant and carer recruitment

Intervention Arm (NAT-C plus Usual Care)

The NAT-C comprises five sections: priority referral for further assessment, patient wellbeing, ability of carer or family to care for patient, carer/family wellbeing and resulting referrals (if required). Clinicians will be encouraged to use the tool as an *aide memoire*, conducting a holistic patient assessment as usual, but referring to the NAT-C to ensure all domains are addressed during a consultation. The NAT-C will be completed using either the electronic medical record template

For peer review only - http://bmjopen.bmj.com/site/about/guidelines.xhtml

(EMIS, SystmOne) or on paper. Completed paper copies of the NAT-C will be uploaded to the patient record.

At least two clinicians per practice will be trained to use the NAT-C either face to face, via webinar or online using a training package piloted during feasibility work.

Participating patients at intervention arm surgeries will be offered a 20 minute appointment or home visit depending on clinical need, guided by a NAT-C trained clinician using the tool within approximately two weeks of study registration. Appointments will take place either at the practice, at patients' homes or remotely via phone or video according to clinical judgement and coronavirus guidelines. Participating carers will be welcome to accompany patients to their appointment, however, the NAT-C allows assessment of carer need through patient response.

Usual care

Usual care is defined as management normally provided for patients with cancer registered at the general practice concerned.[29]

Data collection

Required data, assessment tools, collection time points and processes are summarised in Table 1.

Table 1: Summary of assessments[a]

Participant Assessment (including who is involved)	TIMELINE (months post-randomisation)			
	Baseline	1	3	6
**Eligibility and Consent**				
Consent (P, C, R)	X			
Eligibility (assessed by clinician, R)	X			
**Background and demographics**				
General Demographics (P, C, R)	X			
Cancer Demographics (R - case notes)	X			
Co-morbidities (R - case notes)	X			
**Follow-up data** (collected from case notes)				
Survival status (R)	Ongoing and at the overall end of the trial			
Related Unexpected Serious Adverse Events (R)	Ongoing			
NAT-C Intervention (R)	One month post participant registration			
Usual Care Data (R)	X	X	X	X
**Pre-questionnaire** (phone call at 1, 3, 6 months)				
Performance status (AKPS)	X	X	X	X
COVID status	X	X	X	X
**Participant Questionnaire Booklet** (Self-Completion with researcher support if needed)				
Unmet needs (SCNS-SF34)	X	X	X	X
Symptoms (ESAS-r)	X	X	X	X
Mood and Quality of Life (EORTC QLQ-C15-PAL)	X	X	X	X
EQ-5D-5L	X	X	X	X
ICECAP-SCM	X	X	X	X
Health Care Resource Use (including usual care data and referrals)	X	X	X	X
**Carer Questionnaire Booklet**				

For peer review only - http://bmjopen.bmj.com/site/about/guidelines.xhtml

(Self-Completion with researcher support if needed)				
Carer Experience Scale (CES)	X	X	X	X
Carer wellbeing and burden (ZBI-12)	X	X	X	X

[a] P, participant; C, carer-giver; R, researcher.

Baseline assessments

Clinical data including co-morbidities, cancer stage and treatments will be collected at baseline by the research nurse from the participant's medical record. Demographic information will be collected on participants, including age, sex, participant ethnicity, and living arrangements, during the researcher baseline discussion. For carers, age, sex, relationship status and living arrangements will be collected.

Participant questionnaires

Self-reported participant and carer outcome measures will be collected via questionnaires at baseline, one month and three months post-registration. Questionnaires will also be collected at six months for participants and carers registered before 3 months prior to the end of participant recruitment.

Participants will be able to complete questionnaires using paper forms sent by post, online via REDCap or with a researcher over the phone or face-to-face, as appropriate. Only CTRU data and statistical staff will have direct access to the dataset.

Researchers will telephone participants to confirm questionnaire receipt and assess and collect Performance (AKPS) and COVID-19 status.

Intervention data collection

A research nurse will collect information on NAT-C intervention delivery and content, including the timing, duration, mode of delivery, referrals and subsequent appointments from the participant's medical record.

Safety data collection

In this population, it is expected that episodes of acute illness, infection, new medical problems and deterioration of existing medical problems will occur and could result in prolonged hospitalisation, hospital re-admission, significant or permanent disability or incapacity, or death.

Only serious adverse events fulfilling the definition of a Related Unexpected Serious Adverse Event (RUSAE) resulting from administration of any research procedure, and participant deaths during the trial period, will be recorded. Survival status of participants will be ascertained by research nurses from general practices ahead of sending study follow-up questionnaires.

Deaths

The date and cause of all deaths occurring during the trial period (to last participants 3 month follow-up assessment) will be collected by the researcher from participant's medical record.

STATISTICAL CONSIDERATIONS

Sample Size

For peer review only - http://bmjopen.bmj.com/site/about/guidelines.xhtml

The study has been powered to detect improvement in patients' level of unmet need as measured by proportion of patients reporting at least one moderate or high need in domains of the SCNS-SF34.[30]

Assuming that the proportion of patients with an unmet need on any SCNS-SF34 domain will be similar to that observed pre-intervention by Waller 2012[3]: 64%, then a sample size of 1080 patients recruited from approximately 54 general practices (540 patients, 27 practices per arm), will provide 85% power with a 5% significance level to detect a relative difference of 22% in the proportion of patients with an unmet need. This is an absolute difference of 14%, from 64% to 50%.

The sample size assumes: a 20% loss to follow up rate by 3 months, to account for eligible patients who are, or are nearing, end of life; an Intra-cluster Correlation Coefficient (ICC) of 0.05; an average general practice size of 20; and an adjustment to account for variable practice sizes of 4-40. Given heterogeneity in the design of palliative care services and availability of resources through general practices, and median ICCs reported for outcome variables (0.03) and primary care settings (0.045), an ICC of 0.05 will be used.[31]

Internal pilot and progression criteria

The internal pilot will end 12 months from recruitment of the first general practice. Data from participants in the internal pilot will be included in the main study analysis.

Progression criteria for recruitment are shown in Table 2, based on a traffic-light system of green (go), amber (review) and red (stop), and has been agreed by an independent Trial Steering Committee (TSC) and funder. The TSC will be provided with descriptive data, presented by arm and by general practice to assess internal pilot progression criteria, adherence to the intervention and follow-up, and selection bias at approximately 12 months after the start of the recruitment to inform a decision on continuation of the trial. The internal pilot will not lead to any changes to data collection or the intervention and data from participants in the internal pilot will be included in the main study analysis.

Table 2. Progression criteria for internal pilot

Criteria	Green (go)	Amber (review)	Red (stop)
**Recruitment**<br>General practices assessed at 12 months	80% open (≥43)	50% to 80% open (27-42)	<50% open (<27)
**Recruitment**<br>Participants per month assessed at 12 months (target after 3 months: 60pm)	≥80% (≥48)	50% to 80% (30-47)	<50% (<30)

Statistical analysis

There are no planned interim analyses; outcome data will be analysed once only. All analyses will be conducted on the Intent-to-Treat (ITT) population, in which all general practices and participants will be included in the analysis according to the group which the GP practice was randomised, and

For peer review only - http://bmjopen.bmj.com/site/about/guidelines.xhtml

regardless of non-adherence to the intervention or withdrawal from the study. A two-sided 5% significance level will be used for statistical endpoint comparisons.

The flow of patients and general practices through the trial will be presented in a Consolidated Standards of Reporting Trials (CONSORT) diagram.

As appropriate for cluster trials recruiting participants after randomisation[32], statistical testing of baseline participant data will be at the end of the internal pilot and at the end of the study to assess for selection bias.

Analyses of primary (overall unmet need) and secondary outcomes (unmet needs, severity of symptoms, quality of life, carer wellbeing and burden) will use multi-level logistic or linear regression (as appropriate) with participants nested within general practices, and general practices treated as a random effect. The model will be adjusted for the following fixed effects: GP practice-level stratification factors, important participant-level covariates (e.g. baseline unmet need, age, sex, cancer status, baseline performance status), and other relevant known predictors of outcome. Results will be expressed as point estimates, p-values, ICCs and 95% confidence intervals.

Reasons for attrition and missing participant data will be summarised and mechanisms for missing data we explored according to participant characteristics, intervention and control groups.[33] To conduct analysis on the ITT population, missing data will be multiply imputed at individual participant level under the missing at random assumption. Sensitivity analyses of the primary endpoint will be conducted to assess impact of missing data, choice of imputation model and missing at random assumption.

Quantitative summaries for AKPS score and corresponding change from baseline will be presented at baseline and month 1, 3, and 6 by treatment group. Intervention delivery will be summarised overall and by general practice to evaluate uptake of the NAT-C, adherence to the processes and quality of intervention delivery.

ECONOMIC EVALUATION

Within-trial health economic evaluation will be undertaken to assess cost-effectiveness of NAT-C vs. usual care. The cost-utility analysis will be conducted alongside the trial and follow National Institute for Health and Clinical Excellence (NICE) reference case for health technology appraisals.[34] The main health outcome will be QALYs based on the EQ-5D-5L (base case). Supplementary analyses will estimate cost per improvement in ICECAP-SCM and CES.

We will fully cost intervention delivery and measure service utilisation using a bespoke Resource Use Questionnaire (RUQ) and measure outcomes using the EQ-5D-5L, ICEpop CAPability Supportive Care Measure (ICECAP-SCM) and CES at one, three and six months.

A patient-completed RUQ will gather data on community-based (e.g. contact with GPs, nurses and physiotherapists/occupational therapists), specialist palliative care (hospice, hospital or community) and hospital-based (e.g. A&E visits and hospital attendances) healthcare resource utilisation at follow-up. Participants will be given a diary planner to keep to note any health care attendances to facilitate completion of the RUQ. Costs will be estimated using UK NHS reference unit costs, data from the Personal Social Services Research Unit (PSSRU) and British National Formulary. The primary

For peer review only - http://bmjopen.bmj.com/site/about/guidelines.xhtml

perspective is the health and personal social service provider but a secondary analysis will adopt a wider perspective to incorporate costs and productivity loss incurred by patients and carers.

Results will be presented as incremental cost effectiveness ratio (ICERs). Results will also be presented as expected net monetary benefit and cost effectiveness acceptability curves (CEAC) based on non-parametric bootstrapping.[35] The analysis will employ regression models to adjust for baseline imbalances and account for the correlation between costs and QALYs.[36] The analysis will assume a willingness to pay threshold of £20,000 per incremental QALY with ICERs below this value indicating cost effectiveness.

PROCESS EVALUATION

A mixed-methods sub-study will use Normalisation Process Theory (NPT) to structure data collection and analysis of: 1) implementation of the NAT-C in trial general practices 2) clinicians' and staff perspectives on the usefulness and effectiveness of the NAT-C, how this relates to usual care and how, if effective, the NAT-C could be implemented nationwide.

NPT is a well-established framework for understanding the dynamics involved in implementing, embedding and integrating a new intervention. We will draw upon quantitative and qualitative elements to identify issues related to implementation in terms of 1) a quantitative NPT survey to elicit the views of clinicians who have undergone NAT-C training and 2) qualitative interviews/focus groups with general practice staff, clinicians and external stakeholders with key roles in health policy and commissioning, relevant to cancer care in primary care.

Normalization MeAsure Development Questionnaire (NoMAD) survey

The NPT survey (NoMAD instrument) is a 23-item instrument for measuring implementation processes from the perspective of professionals directly involved in the work of implementing complex interventions. During feasibility testing, we adapted the NoMAD instrument in to a 17-point checklist to specifically address the NAT-C. Clinicians will be invited to complete the NoMAD survey either on paper or online following completion of NAT-C training (Survey 1). Using results from Survey 1, emerging qualitative findings and experiences, the NoMAD will be adapted to include questions regarding emerging issues and concerns. At the end of a practices' involvement with the study, clinicians who have used the NAT-C will be asked to complete the adapted NoMAD survey (Survey 2).

Clinicians will be asked questions on a Likert scale in relation to issues such as: attitudes to the NAT-C, NAT-C training and implementation concerns. Completion of the survey will imply informed consent. Data collection and management for surveys 1 and 2 will be delivered by the University of Hull. All survey data will be anonymised.

Interviews and focus groups

Opinion regarding NAT-C training, the role and place of the NAT-C within routine practice will be sought from clinicians who received NAT-C training and experts from a range of stakeholder groups (e.g. local commissioning groups, general practice federations, the National Cancer Research Institute's primary care group, Royal College of GPs, and Macmillan). Semi-structured interviews and focus groups using *a priori* topic guides (either phone/video conferencing or face-to-face, as appropriate) will be conducted at various time-points post-NAT-C use and up to the end of study. Interviews/focus groups with clinicians and key stakeholders will focus upon structural and policy

For peer review only - http://bmjopen.bmj.com/site/about/guidelines.xhtml

issues relevant to potential implementation of the NAT-C in general practices nationwide, should trial results be positive.

Maximum variation purposive sampling will be used to optimise exploration of a range of clinicians, practice staff and key stakeholder perspectives. An initial purposive sampling grid for clinicians (profession, years of clinical practice, randomisation strata) will be expanded with further criteria identified from implementation study survey responses.

A sample of 15-20 clinicians and general practice staff and 10 -15 experts from a range of stakeholders will be sought through interviews or focus group.

Potential interviewees will be provided with a Study Invitation, a Study Information Sheet and asked to provide informed written consent prior to study procedures. All interviews and focus group discussions will be audio-recorded.

NoMAD Survey analysis

Free-text responses in survey 1 will be monitored by the implementation study researcher to enable rapid feedback to inform subsequent training at other sites.[37]

Once all surveys 1 and 2 are completed, free text responses will be subject to thematic analysis and descriptive statistics will be used to analyse Likert scale responses including: 1) the extent to which the intervention fits with current practice in relation to the components of NPT; 2) the potential relevance of the NAT-C to individuals' roles; 3) adequacy of NAT-C training; and 4) clinician attitudes to the NAT-C at baseline and at the end of the trial from Survey 1 and 2.

Interview/focus group analysis

Qualitative data will be analysed using thematic analysis[37], informed by NPT, relating to: how clinicians understand the intervention (coherence); how they engage with it (cognitive participation); enact it (collective action); and appraise its effects (reflexive monitoring).[38] The end of trial analysis will develop themes in relation to how the NAT-C could be implemented in primary care nationally, should trial be results be positive. Transcripts will be coded line by line.

Synthesis with intervention uptake data

We will synthesise key aspects of process evaluation data, with effectiveness of the NAT-C within clusters according to randomisation strata, to improve understanding using NPT about how and if the NAT-C should be implemented into clinical practice using Critical Interpretative Synthesis (CIS).[39]

Kirkpatrick's model for training evaluation will be used to evaluate NAT-C training in terms of: reaction to the training, learning and skills improvement, behavioural change and results.[40] Reaction will be assessed by responses to NoMAD surveys and interview. Learning and behavioural change will be evaluated through qualitative data.

Trial organization and governance

CANAssess is sponsored by the University of Hull (UoH) coordinated by Leeds CTRU and UoH. The sponsor had no direct input in to the design or conduct of the study. The Trial Management Group consists (TMG) of co-applicants, trial coordinators, four GP-hub leads and a public-patient representative. The TMG is responsible for clinical set-up, on-going management, promotion of the

For peer review only - http://bmjopen.bmj.com/site/about/guidelines.xhtml

trial, and for the interpretation and publishing of the results. A TSC will meet annually and on request to provide independent oversight of the trial and reports to the Sponsor.

A Data Monitoring and Ethics Committee (DMEC) is not needed due to the nature of the study. The TSC will adopt a safety monitoring role, with the constitution of a sub-committee to review safety issues where necessary.

Patient and public involvement

An experienced lay representative was part of our funding application. She also reviewed and edited public-facing study documentation, and sits on our TMG, with public-patient involvement as a standing item. A further lay representative forms part of our TSC.

Ethics and Dissemination

Dissemination

If trial results are positive, the NAT-C has the potential to become the gold standard cancer care delivery in primary care as the only valid tool subjected to formal effectiveness testing.

Findings will be presented and discussed at a final dissemination meeting, to which a wide range of stakeholders will be invited, including trial clinicians, participants and those involved in the stakeholder engagement.

Results of the study will be published in peer-review publications and will be presented at national and international conferences. A lay summary of our findings will be published on study and organizational websites, sent to participating general practices and will be accessible to participants.

Ethical considerations

The trial received ethical approval from the London-Surrey REC (20/LO/0312). Any future amendments to the trial will be submitted to the REC and participants will be informed of any changes which may affect them.

For peer review only

For peer review only - http://bmjopen.bmj.com/site/about/guidelines.xhtml

Impact of COVID-19

The COVID-19 outbreak in England occurred just as ethical approval for the study had been obtained and the process of site identification had begun. We halted site identification and adapted the trial processes to allow remote intervention delivery as per practice procedure for remote consultations, telephone consent and data collection, and online patient study responses and online completion of follow-up questionnaires. Amidst concerns that patient recruitment may be affected by social distancing measures, the Leeds CTRU also highlighted how their secure online computer systems would allow online informed consent provision and data collection. We therefore submitted an amendment to allow all study activity to be completed remotely through phone or video-conference.

Trial status

Following COVID-related delays, the trial team is in place, incorporating employed trial-specific research nurses and Clinical Research Network (CRN) support. Recruitment of GP practices and participants is underway. Our first study site was opened for recruitment on 21.10.2020 and we now have seven general practices recruiting participants. The first participant was recruited on 01/12/2020. As of25.01.2021 we have 27 general practices open to recruitment and have recruited 333 patient participants and 102 carer participants. . This manuscript has been prepared in accordance with study protocol v.3, 24.06.2020. A copy of the full protocol is available on request from Dr Joseph Clark.

Trial registration

ISRCTN15497400, registered 07/04/2020

Funding

This work was supported by Yorkshire Cancer Research, grant number: H423

Authors' contributions

All authors meet the International Committee of Medical Journal Editors (ICMJE) criteria for authorship and contributed to the work presented as follows: conception and design of the trial (JC, AW-H, DM, RF, SW, JD, TM, AF, PB, EM, MJ), development of data analysis methods (AF, BC, DM, AW-H), process evaluation methods (JC/MJ). JC produced a first draft the manuscript, after which all authors commented and provided edits ahead of finalisation. All authors approved the final draft and agree to be held accountable for all aspects of the work by ensuring that questions related to the accuracy and integrity of the work are appropriately investigated and resolved.

Correspondence should be directed to: Dr Joseph Clark, e: joseph.clark@hyms.ac.uk

Competing interests

All authors declare no competing interests.

References

---

[1] Ruijs, C.D., Kerkhof, A.J., van der Wal, G. et al. Symptoms, unbearability and the nature of suffering in terminal cancer patients dying at home: a prospective primary care study. *BMC Fam Pract* 14, 201 (2013). https://doi.org/10.1186/1471-2296-14-201

For peer review only - http://bmjopen.bmj.com/site/about/guidelines.xhtml

[2] Adams E, Boulton M and Rose P et al. Views of cancer care reviews in primary care: a qualitative study. *BJGP,* 2011;61(585):e173-e183.
[3] Waller A, Girgis A, Johnson C, et al. Improving outcomes for people with progressive cancer: interrupted time series trial of a needs assessment intervention. *J Pain Symptom Manage* 2012;43:569–81.
[4] Homsi J, Walsh D, Rivera N, Rybicki LA, Nelson KA, Legrand SB, Davis M, Naughton M, Gvozdjan D and Pham H. Symptom evaluation in palliative medicine: patient report vs systematic assessment, *Support Care Cancer,* 2006;14(5):444-53.
[5] Carlson LE, Waller A, Mitchell AJ. Screening for distress and unmet needs in patients with cancer: review and recommendations. J Clin Oncol. 2012;30(11):1160-77.
[6] Department of Health and Social Care. Living With and Beyond Cancer: Taking Action to Improve Outcomes 2013 [Available from: https://assets.publishing.service.gov.uk/government/uploads/system/uploads/attachment_data/file/181054/9333-TSO-2900664-NCSI_Report_FINAL.pdf.
[7] Foy R, Leaman B, McCrorie C, Petty D, House A, Bennett M, Carder P, Faulkner S, Glidewell L, West R. Prescribed opioids in primary care: cross sectional and longitudinal analyses of influence of patient and practice characteristics. BMJ Open 2016;6:e010276. doi:10.1136/bmjopen-2015-010276.
[8] Tamburini M, Gangeri L, Brunelli C et al. Assessment of hospitalised cancer patients' needs by the Needs Evaluation Questionnaire. *Ann Oncol,* 2000; 11(1):31-37.
[9] Davidson PM, Paull G, Introna K et al. Integrated, collaborative palliative care in heart failure: the St. George Heart Failure Service experience 1999-2002. *J Cardiovasc Nurs,* 2004; 19(1):68-75.
[10] Deeken JF, Taylor KL, Mangan P, Yabroff KR, Ingham JM. Care for the caregivers: a review of self-report instruments developed to measure the burden, needs, and quality of life of informal caregivers. *J Pain Symptom Manage,* 2003; 26(4):922-953.
[11] Wen KY, Gustafson DH. Needs assessment for cancer patients and their families. *Health Qual Life Outcomes,* 2004; 2:11.
[12] Girgis A, Johnson C, Currow DC, et al. Palliative Care Needs Assessment Guidelines. The Centre for Health Research & Psycho-oncology. 2006. Newcastle, NSW.
[13] Girgis A, Johnson C, Currow D, Waller A, Kristjanson L, Mitchell G, et al. Palliative care needs assessment guidelines. 2006
[14] Groenvold M, Petersen MA, Aaronson NK, Arraras JI, Blazeby JM, Bottomley A, et al. EORTC QLQ-C15-PAL: the new standard in the assessment of health-related quality of life in advanced cancer? Palliat Med. 2006;20(2):59-61.
[15] Groenvold M, Petersen MA, Aaronson NK, Arraras JI, Blazeby JM, Bottomley A, et al. The development of the EORTC QLQ-C15-PAL: a shortened questionnaire for cancer patients in palliative care. Eur J Cancer. 2006;42(1):55-64.
[16] Osse BHP, Vernooij-Dassen MJFJ, de Vree BPW, Schade E, Grol RPTM. Assessment of the need for palliative care as perceived by individual cancer patients and their families - A review of instruments for improving patient participation in palliative care. Cancer. 2000;88(4):900-11.
[17] Allgar VL, Chen H, Richfield E, Currow D, Macleod U, Johnson MJ. Psychometric Properties of the Needs Assessment Tool-Progressive Disease Cancer in U.K. Primary Care. *J Pain Symptom Manage*, 2018 Oct;56(4):602-612. doi: 10.1016/j.jpainsymman.2018.07.002. Epub 2018 Aug 7. PMID: 30009964.
[18] Clark J, Amoakwa E and Wright-Hughes A et al. A cluster randomised trial of a Needs Assessment Tool for adult Cancer patients and their carers (NAT-C) in primary care: A feasibility study. *Plos One,* 2021; https://doi.org/10.1371/journal.pone.0245647.
[19] Boyes A, Girgis A, Lecathelinais C. Brief assessment of adult cancer patients' perceived needs: development and validation of the 34-item Supportive Care Needs Survey (SCNS-SF34). J Eval Clin Pract. 2009;15(4):602-6.
[20] Abernethy AP, Shelby-James T, Fazekas BS, Woods D, Currow DC. The Australia-modified Karnofsky Performance Status (AKPS) scale: a revised scale for contemporary palliative care clinical practice [ISRCTN81117481]. BMC Palliat Care. 2005;4:7.
[21] Services AH. Guidelines for using the revised Edmonton Symptom Assessment System (ESAS-r) [Website]. 2010 [Available from: http://www.palliative.org/NewPC/_pdfs/tools/3C7%20ESAS-r%20guidelines%20Aug%2022%202014.pdf.

For peer review only - http://bmjopen.bmj.com/site/about/guidelines.xhtml

[22] Groenvold M, Petersen MA, Aaronson NK, Arraras JI, Blazeby JM, Bottomley A, et al. EORTC QLQ-C15-PAL: the new standard in the assessment of health-related quality of life in advanced cancer? Palliat Med. 2006;20(2):59-61.
[23] Goranitis I, Coast J, Al-Janabi H. An investigation into the construct validity of the Carer Experience Scale (CES). Qual Life Res. 2014;23(6):1743-52.
[24] Higginson IJ, Gao W, Jackson D, Murray J, Harding R. Short-form Zarit Caregiver Burden Interviews were valid in advanced conditions. J Clin Epidemiol. 2010;63(5):535-42
[25] Herdman M, Gudex C, Lloyd A, Janssen M, Kind P, Parkin D, et al. Development and preliminary testing of the new five-level version of EQ-5D (EQ-5D-5L). Qual Life Res. 2011;20(10):1727-36.
[26] Huynh E, Coast J, Rose J, Kinghorn P and Flynn T. Values for the ICECAP-Supportive Care Measure (ICECAP-SCM) for use in economic evaluation at end of life. *Soc Sci Med,* 2017;189:114-128.
[27] NHS Digital. Organisation Data Service 2019 [Available from: https://digital.nhs.uk/services/organisation-data-service.
[28] NHS Digital. NHS Payments to General Practice - England, 2018/19 2019 [Available from: https://digital.nhs.uk/data-and-information/publications/statistical/nhs-payments-to-general-practice/england-2018-19. Accessed 30.10.2020.
[29] NHS England. Standard General Medical Services Contract 2018/19 2019 [Available from: https://www.england.nhs.uk/wp-content/uploads/2019/04/general-medical-services-contract-19-20.pdf.
[30] McElduff P, Boyes A, Zucca A, Girgis AJNCfHR, Psycho-oncology. Supportive Care Needs Survey: A guide to administration, scoring and analysis. Newcastle: Centre for Health Research and Psycho-oncology; 2004.
[31] Campbell MK, Fayers PM, Grimshaw JM. Determinants of the intracluster correlation coefficient in cluster randomized trials: the case of implementation research. Clin Trials. 2005;2(2):99-107.
[32] Bolzern JE, Mitchell A, Torgerson DJ. Baseline testing in cluster randomised controlled trials: should this be done? BMC Med Res Methodol. 2019;19(1):106.
[33]Higginson IJ, Evans CJ, Grande G, et al. . Evaluating complex interventions in end of life care: the MORECare statement on good practice generated by a synthesis of transparent expert consultations and systematic reviews. BMC Med 2013;11:11 10.1186/1741-7015-11-111
[34] National Institute for Health and Clinical Excellence (NICE). Guide to the methods of technology appraisal London: NICE; 2013 [Available from: https://www.nice.org.uk/process/pmg9/resources/guide-to-the-methods-of-technology-appraisal-2013-pdf-2007975843781.
[35] Barton GR, Briggs AH, Fenwick EA. Optimal cost-effectiveness decisions: the role of the cost-effectiveness acceptability curve (CEAC), the cost-effectiveness acceptability frontier (CEAF), and the expected value of perfection information (EVPI). Value in health. 2008 Sep;11(5):886-97.
[36] Willan AR, Briggs AH, Hoch JS. Regression methods for covariate adjustment and subgroup analysis for non-censored cost-effectiveness data. Health economics. 2004 May;13(5):461-75.
[37] Silverman D. Doing Qualitative Research. 3rd Edition ed. London: Sage; 2009.
[38] Normalization Process Theory. Core Propositions of NPT 2019 [Available from: http://www.normalizationprocess.org/theory-behind-npt/core-propositions-of-npt/.
[39] Dixon-Woods M, Cavers D and Agarwal S et al. Conducting a critical interpretive synthesis of the literature on access to healthcare by vulnerable groups. *BMC Med Res Method,* 2006;6@53. doi:10.1186/1471-2288-6-35.
[40] Barr H, Freeth D, Hammick M, Koppel I, Reeves S. Evaluations of interprofessional education: a United Kingdom review for health and social care. 2000. Available from: https://www.caipe.org/resources/publications/barr-h-freethd-hammick-m-koppel-i-reeves-s-2000-evaluations-of-interprofessional-education. 30.10.2020.

For peer review only - http://bmjopen.bmj.com/site/about/guidelines.xhtml

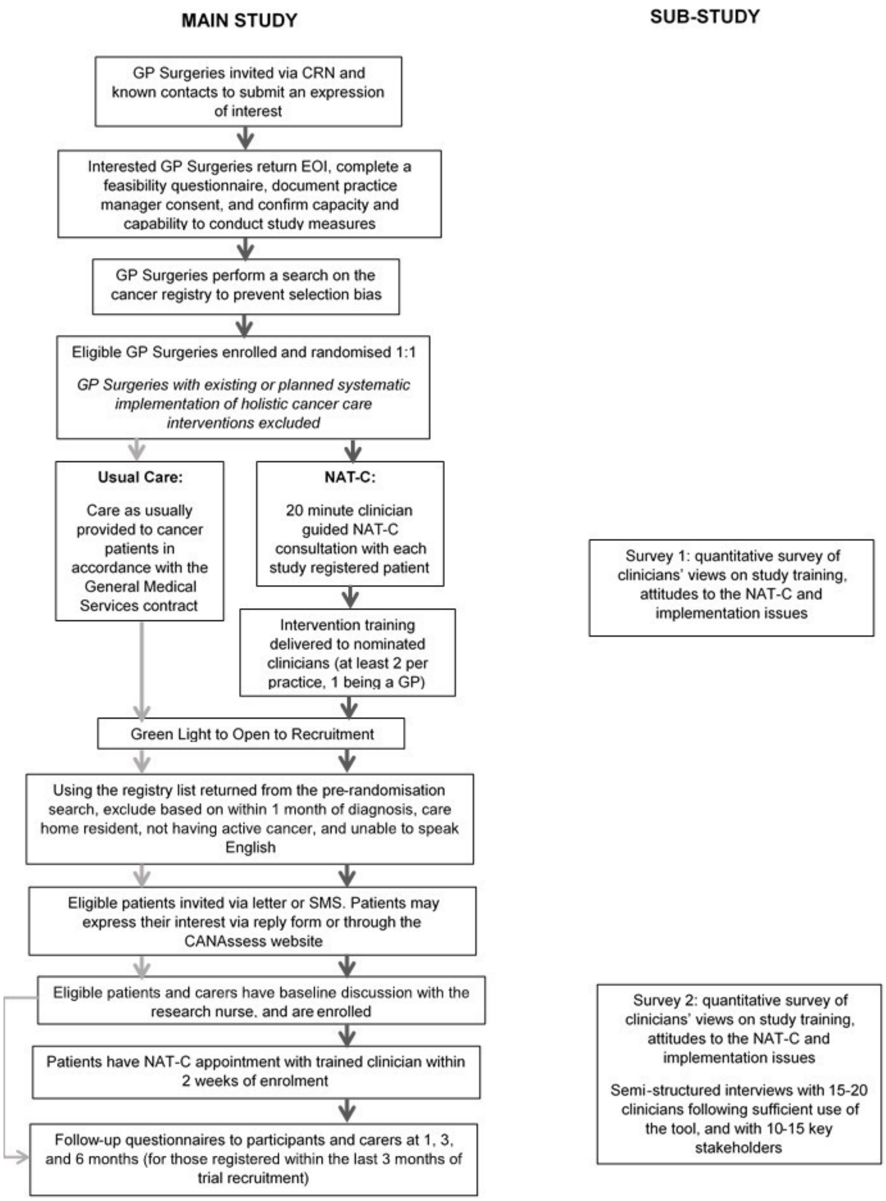

Figure 1: Study Flow Chart

58x79mm (300 x 300 DPI)

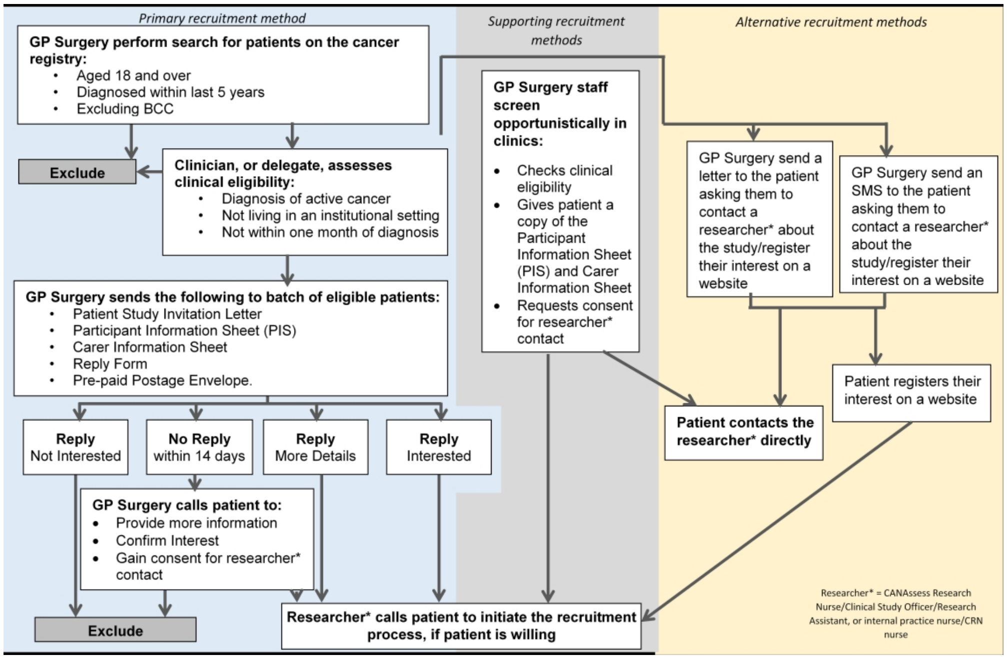

Figure 2. Participant and carer recruitment

536x349mm (300 x 300 DPI)

Supplementary file 1 –Risk of bias assessment

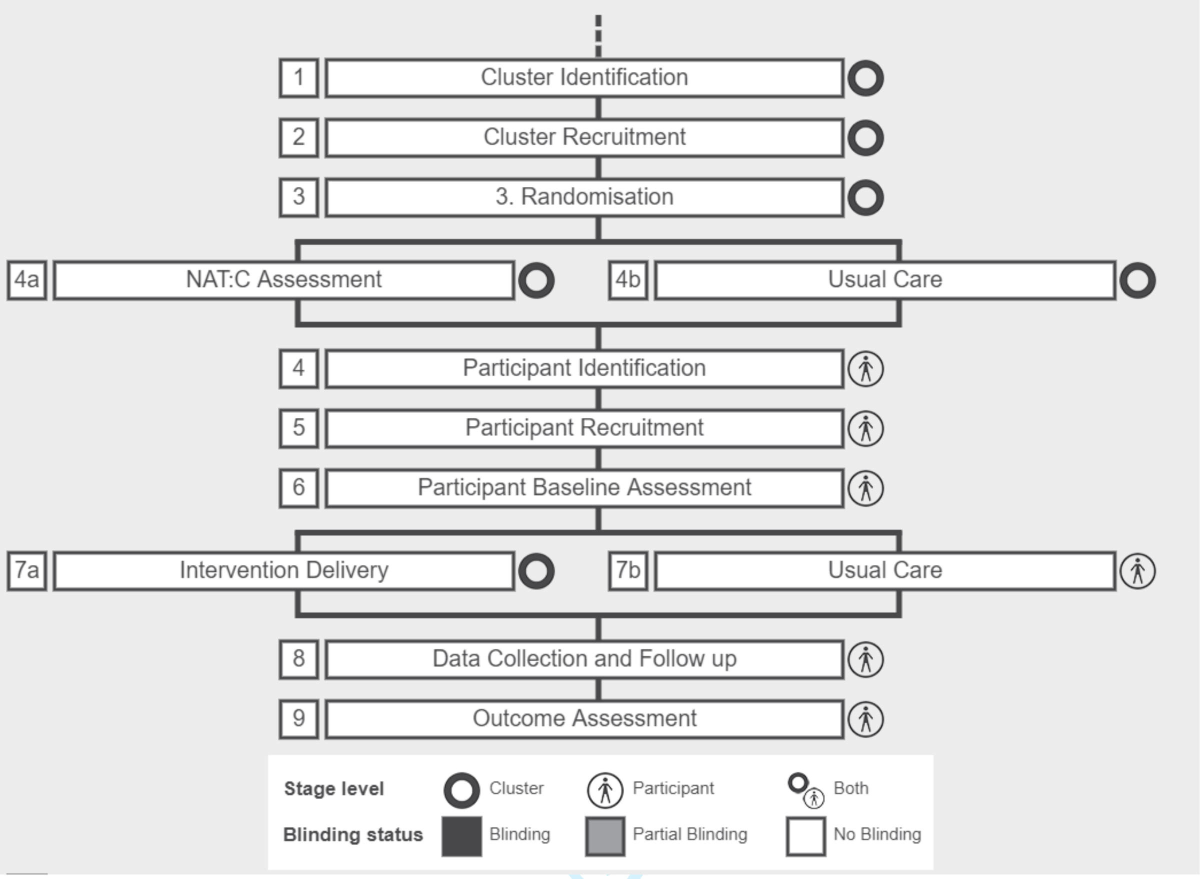

1. Cluster identification

General Practices in Yorkshire and Tyne and Wear will be approached and 54 recruited. Cluster identification will be conducted by four separate 'hubs', located at Leeds, Hull, Sheffield and the North East of England, each co-ordinated by a clinical 'hub-lead'. Practices will be invited to submit an Expression of Interest through relevant Clinical Research Network (CRN) mailing lists and hub lead networks. Practices will be asked to confirm their capacity to deliver the trial and eligibility will be assessed by the research team in terms of local research capacity.

2. Cluster recruitment

General Practices will be eligible unless they: took part in the feasibility study, have implemented or are planning to implement within the duration of the trial a systematic holistic cancer care intervention that overlaps with the NAT-C, or are unable to confirm capacity and capability to deliver the study at their GP Surgery. Practices will provide consent to deliver the study on the terms stated in a Schedule of Events Cost Attribution Template (SoECAT).

3. Randomisation

General practices will be randomised with a 1:1 ratio level by a statistician at the Leeds Clinical Trials Unit. Randomisation will take place post-site initiation. Practices will be randomised to either i) Needs Assessment Tool – Cancer NAT-C) plus Usual Care or ii) Usual Care, stratified by: Locality; Urban or rural area (UK government rural-urban classification based on GP Surgery postcode; List Size: <5000, 5000-10000, >10000 (obtained from NHS digital); A GP training practice (obtained from

site feasibility questionnaire): Yes, No. Practices will be randomised after consent and prior to study training and participant identification. Training will take place either face to face, via video-link or with a piloted online training package.

4. Participant Identification

An administrator or research nurse will conduct a database search for patients with ‘active cancer’ post-randomisation. A date restriction of five years will be applied in terms of date of diagnosis. This will remove historic cancer cases from the results, but may miss patients who have been living with active cancer for more than five years. A further exclusion will remove people with basal cell carcinoma (BCC) using a read code. A clinician will assess participant eligibility, in particular, to confirm a current cancer diagnosis and to confirm capacity to provide informed consent. There is a small risk that clinicians may exclude patients due to stage of illness. The research team will encourage clinicians to give patients at any stage of illness the opportunity to take part. Eligibility will be defined by a clinic and eligibility checks will take place during study monitoring conducted by a trained researcher.

5. Participant Recruitment

Eligible patients will be invited to the study either via letter, SMS or opportunistically at General Practices. All eligible patients will be provided with a Study Invitation Sheet. A Research Nurse will contact patients expressing interest in the study, answer any questions that the patient may and arrange informed written consent. Witnessed informed consent may be taken if a patient is unable to write.

Participating patients will be given the opportunity to nominate a carer if they would like to. Nominated carers will then receive a Carer Information Sheet and a Study Invitation. A Research Nurse will answer any questions that carer may have about the study ahead of arranging informed written consent.

Participants will find out which arm of the trial their practice has been allocated to after providing informed consent.

6. Participant Baseline Assessment

After taking written informed consent, a Research Nurse will help participants to provide baseline information. Demographic information and clinical characteristics will be collected. During a face to face appointment, a research nurse will collect participant: age, sex, cancer type and stage, treatment history, ethnicity, relationship status, living arrangement and accommodation, household income, postcode, the Australian Karnofsky Performance Status (AKPS) and the Charlson Co-morbidity Index. Patients will then be advised regarding their allocation and advised how to proceed win the study.

7a. Intervention Delivery

General Practitioners and clinical nurses will receive training in how to use the NAT:C either face to face or online. Research nurses will not receive intervention training. General practices will then contact patients to arrange a twenty minute needs assessment appointment, to occur within two weeks of informed consent. Clinicians will conduct a twenty-minute needs assessment appointment using the NAT-C. The NAT-C will be available as a template on EMIS and SystmOne and a paper copy will be available to clinicians if required. Patients may attend their needs assessment appointment

For peer review only

with a carer if they would like to. The carer does not have to be participating in the study. Patients will also have access their General Practice as usual.

7b. Usual Care

Patients will have access to their General Practice as usual.

8. Data collection and follow up

Patient participants will be asked to complete follow up questionnaires at one month and three months: the Supportive Care Needs Survey, the AKPS, the revised Edmonton Symptom Assessment System (ESAS-r), the EORTC QLQ-C15-PAL and a bespoke Resource Use Questionnaire (RUQ). Participants will be supported by a research nurse during data collection either face to face or over the phone. Completed NAT:C assessments will be retrieved from the practice clinical record. It will not be possible to blind research nurses to the allocation of General Practices (and therefore patients) during data collection. However, data collection will not be conducted by anybody who has been involved in delivering the intervention. Data collection will be undertaken as close to the stated time points as feasible.

9. Outcome assessment

Primary outcome will be proportion of patients with an unmet need on the SCNS. Analysis will be conducted on an intent to treat basis. Final analysis will be conducted by a senior statistician at the Leeds Clinical Trials Unit and will take place once all participants have completed three month measures.

For peer review only

Delete this line, then print first page on practice-headed paper

CANAssess
Cancer Patients' Needs Assessment in Primary Care

Funded by yorkshire cancer research

CANAssess 2: Cancer Patients' Needs Assessment in Primary Care – A Cluster Randomised Controlled Trial

Participant ID:	Initials:
Date of Birth:	NHS Number:
ISRCTN: 15497400	Principal Investigator:

For peer review only

PARTICIPANT CONSENT FORM

	*Please **initial** each box below*
1. I confirm that I have read and understand the information sheet dated <<INSERT DATE>> (version X.0) for the above study. I have had the opportunity to consider the information, ask questions, and have had these answered satisfactorily	
2. I understand that my participation in this study is voluntary and that I am free to withdraw at any time without my medical care or legal rights being affected.	
3. I understand that if I withdraw from the above study, the data collected from me up until that point will be used in analysing the results of the study.	
4. I agree for my personal details (including name, date of birth, address, postcode, email address, telephone number, NHS number, GP name and GP address) to be securely stored in accordance with the study sponsor guidance (minimum 5 years).	
5. I understand that relevant sections of any of my medical records and/or study data may be looked at by responsible individuals from the research team, the sponsor (University of Hull), Leeds Clinical Trials Research Unit (CTRU), relevant third parties or from regulatory authorities where it is relevant to my taking part in the research. I give permission for these individuals to access my records.	
6. I understand that if during this study my clinical care team determine that I have lost my ability to make my own decisions, I will be withdrawn from the study and no further study information will be collected. The data collected from me up until that point will be used in analysing the results of the study.	
7. I consent to the secure transfer, storage and use of paper and electronic personal information, for the purposes of this study to the CTRU, or relevant third parties. I understand that any information that could identify me will be kept strictly confidential and that no personal information will be included in the study report or other publication.	
8. I agree to a copy of this Consent Form being sent to the CTRU.	

For peer review only - http://bmjopen.bmj.com/site/about/guidelines.xhtml

Delete this line, then print first page on practice-headed paper

9. I agree to my General Practitioner being informed of my participation in this study and being provided with a copy of this consent form. I understand that my GP will be advised of any significant information relating to my health that comes to light.	
10. I agree to take part in the study.	

Optional:

Even if you agree to take part in this study, you do not have to agree to this statement. Please initial next to 'yes' **or** 'no'.

11. I agree that the information collected about me may be used to support other research in the future, and may be shared anonymously with other researchers.	Yes	
	No	

Method of Consent:

☐ **Telephone Consent:**

Researcher:

I have explained the study and read each consent statement to the above named patient. He/she has indicated his/her willingness to participate and agreed to each compulsory statement, so I have initialled and signed on their behalf.

Signature……………………………………..……………………………………

Name (block capitals)……………………………………………………………

Date……………………………………………….…………………………………

☐ **Face-To-Face Consent:**

Patient:

Signature……………………………………………………………………………

Name (block capitals)………………………………………………….……………

Date……………………………………………….…………………………………

For peer review only

For peer review only - http://bmjopen.bmj.com/site/about/guidelines.xhtml

Delete this line, then print first page on practice-headed paper

Researcher:

I have explained the study to the above named patient and he/she has indicated his/her willingness to participate.

Signature………………………………………..…………………………………

Name (block capitals)……………………………………………….……………

Date………………………………………………..……………………………………

Witness/Translator:

I have completed this consent form on behalf of the person named above who has freely given their verbal consent to participate.

Signature………………………………………..…………………………………

Name (block capitals)……………………………………………….……………

Date………………………………………………..……………………………………

(1 copy for patient; 1 for the CTRU; 1 held in patient notes, original stored in Investigator Site File)

For peer review only

For peer review only - http://bmjopen.bmj.com/site/about/guidelines.xhtml

STANDARD PROTOCOL ITEMS: RECOMMENDATIONS FOR INTERVENTIONAL TRIALS

SPIRIT 2013 Checklist: Recommended items to address in a clinical trial protocol and related documents*

Section/item	Item No	Description	Page Number on which item is reported
**Administrative information**			
Title	1	Descriptive title identifying the study design, population, interventions, and, if applicable, trial acronym	3
Trial registration	2a	Trial identifier and registry name. If not yet registered, name of intended registry	16
	2b	All items from the World Health Organization Trial Registration Data Set	3,4, 7, 10, 14, 16
Protocol version	3	Date and version identifier	16
Funding	4	Sources and types of financial, material, and other support	16
Roles and responsibilitie s	5a	Names, affiliations, and roles of protocol contributors	16
	5b	Name and contact information for the trial sponsor	16
	5c	Role of study sponsor and funders, if any, in study design; collection, management, analysis, and interpretation of data; writing of the report; and the decision to submit the report for publication, including whether they will have ultimate authority over any of these activities	16
	5d	Composition, roles, and responsibilities of the coordinating centre, steering committee, endpoint adjudication committee, data management team, and other individuals or groups overseeing the trial, if applicable (see Item 21a for data monitoring committee)	16
**Introduction**			

For peer review only - http://bmjopen.bmj.com/site/about/guidelines.xhtml

Background and rationale	6a	Description of research question and justification for undertaking the trial, including summary of relevant studies (published and unpublished) examining benefits and harms for each intervention	3-4
	6b	Explanation for choice of comparators	9
Objectives	7	Specific objectives or hypotheses	4-5
Trial design	8	Description of trial design including type of trial (eg, parallel group, crossover, factorial, single group), allocation ratio, and framework (eg, superiority, equivalence, noninferiority, exploratory)	4
**Methods: Participants, interventions, and outcomes**			
Study setting	9	Description of study settings (eg, community clinic, academic hospital) and list of countries where data will be collected. Reference to where list of study sites can be obtained	5
Eligibility criteria	10	Inclusion and exclusion criteria for participants. If applicable, eligibility criteria for study centres and individuals who will perform the interventions (eg, surgeons, psychotherapists)	5, 6, 13, 14
Interventions	11a	Interventions for each group with sufficient detail to allow replication, including how and when they will be administered	9,10
	11b	Criteria for discontinuing or modifying allocated interventions for a given trial participant (eg, drug dose change in response to harms, participant request, or improving/worsening disease)	NA
	11c	Strategies to improve adherence to intervention protocols, and any procedures for monitoring adherence (eg, drug tablet return, laboratory tests)	4,5
	11d	Relevant concomitant care and interventions that are permitted or prohibited during the trial	9
Outcomes	12	Primary, secondary, and other outcomes, including the specific measurement variable (eg, systolic blood pressure), analysis metric (eg, change from baseline, final value, time to event), method of aggregation (eg, median, proportion), and time point for each outcome. Explanation of the clinical relevance of chosen efficacy and harm outcomes is strongly recommended	4,5

For peer review only - http://bmjopen.bmj.com/site/about/guidelines.xhtml

Participant timeline	13	Time schedule of enrolment, interventions (including any run-ins and washouts), assessments, and visits for participants. A schematic diagram is highly recommended (see Figure)	9,10
Sample size	14	Estimated number of participants needed to achieve study objectives and how it was determined, including clinical and statistical assumptions supporting any sample size calculations	11
Recruitment	15	Strategies for achieving adequate participant enrolment to reach target sample size	6,7
**Methods: Assignment of interventions (for controlled trials)**			
Allocation:			
Sequence generation	16a	Method of generating the allocation sequence (eg, computer-generated random numbers), and list of any factors for stratification. To reduce predictability of a random sequence, details of any planned restriction (eg, blocking) should be provided in a separate document that is unavailable to those who enrol participants or assign interventions	6
Allocation concealment mechanism	16b	Mechanism of implementing the allocation sequence (eg, central telephone; sequentially numbered, opaque, sealed envelopes), describing any steps to conceal the sequence until interventions are assigned	6
Implementation	16c	Who will generate the allocation sequence, who will enrol participants, and who will assign participants to interventions	6
Blinding (masking)	17a	Who will be blinded after assignment to interventions (eg, trial participants, care providers, outcome assessors, data analysts), and how	NA
	17b	If blinded, circumstances under which unblinding is permissible, and procedure for revealing a participant's allocated intervention during the trial	NA
**Methods: Data collection, management, and analysis**			

Data collection methods	18a	Plans for assessment and collection of outcome, baseline, and other trial data, including any related processes to promote data quality (eg, duplicate measurements, training of assessors) and a description of study instruments (eg, questionnaires, laboratory tests) along with their reliability and validity, if known. Reference to where data collection forms can be found, if not in the protocol	9,10
	18b	Plans to promote participant retention and complete follow-up, including list of any outcome data to be collected for participants who discontinue or deviate from intervention protocols	9, 10
Data management	19	Plans for data entry, coding, security, and storage, including any related processes to promote data quality (eg, double data entry; range checks for data values). Reference to where details of data management procedures can be found, if not in the protocol	7
Statistical methods	20a	Statistical methods for analysing primary and secondary outcomes. Reference to where other details of the statistical analysis plan can be found, if not in the protocol	11,12,
	20b	Methods for any additional analyses (eg, subgroup and adjusted analyses)	12,13,14
	20c	Definition of analysis population relating to protocol non-adherence (eg, as randomised analysis), and any statistical methods to handle missing data (eg, multiple imputation)	12
**Methods: Monitoring**			
Data monitoring	21a	Composition of data monitoring committee (DMC); summary of its role and reporting structure; statement of whether it is independent from the sponsor and competing interests; and reference to where further details about its charter can be found, if not in the protocol. Alternatively, an explanation of why a DMC is not needed	15
	21b	Description of any interim analyses and stopping guidelines, including who will have access to these interim results and make the final decision to terminate the trial	11,12

For peer review only - http://bmjopen.bmj.com/site/about/guidelines.xhtml

Harms	22	Plans for collecting, assessing, reporting, and managing solicited and spontaneously reported adverse events and other unintended effects of trial interventions or trial conduct	11
Auditing	23	Frequency and procedures for auditing trial conduct, if any, and whether the process will be independent from investigators and the sponsor	11
**Ethics and dissemination**			
Research ethics approval	24	Plans for seeking research ethics committee/institutional review board (REC/IRB) approval	15
Protocol amendments	25	Plans for communicating important protocol modifications (eg, changes to eligibility criteria, outcomes, analyses) to relevant parties (eg, investigators, REC/IRBs, trial participants, trial registries, journals, regulators)	15
Consent or assent	26a	Who will obtain informed consent or assent from potential trial participants or authorised surrogates, and how (see Item 32)	7
	26b	Additional consent provisions for collection and use of participant data and biological specimens in ancillary studies, if applicable	NA
Confidentiality	27	How personal information about potential and enrolled participants will be collected, shared, and maintained in order to protect confidentiality before, during, and after the trial	10
Declaration of interests	28	Financial and other competing interests for principal investigators for the overall trial and each study site	16
Access to data	29	Statement of who will have access to the final trial dataset, and disclosure of contractual agreements that limit such access for investigators	10
Ancillary and post-trial care	30	Provisions, if any, for ancillary and post-trial care, and for compensation to those who suffer harm from trial participation	NA
Dissemination policy	31a	Plans for investigators and sponsor to communicate trial results to participants, healthcare professionals, the public, and other relevant groups (eg, via publication, reporting in results databases, or other data sharing arrangements), including any publication restrictions	15

For peer review only

	31b	Authorship eligibility guidelines and any intended use of professional writers	16
	31c	Plans, if any, for granting public access to the full protocol, participant-level dataset, and statistical code	16
**Appendices**			
Informed consent materials	32	Model consent form and other related documentation given to participants and authorised surrogates	NA
Biological specimens	33	Plans for collection, laboratory evaluation, and storage of biological specimens for genetic or molecular analysis in the current trial and for future use in ancillary studies, if applicable	NA

*It is strongly recommended that this checklist be read in conjunction with the SPIRIT 2013 Explanation & Elaboration for important clarification on the items. Amendments to the protocol should be tracked and dated. The SPIRIT checklist is copyrighted by the SPIRIT Group under the Creative Commons "Attribution-NonCommercial-NoDerivs 3.0 Unported" license.

For peer review only

For peer review only - http://bmjopen.bmj.com/site/about/guidelines.xhtml

TIDieR
Template for Intervention Description and Replication

The TIDieR (Template for Intervention Description and Replication) Checklist*:

Information to include when describing an intervention and the location of the information

Item number	Item	Where located ** Primary paper (page or appendix number)	Other † (details)
	**BRIEF NAME**		
**1.**	Provide the name or a phrase that describes the intervention.	______4,5____ __	______________
	**WHY**		
**2.**	Describe any rationale, theory, or goal of the elements essential to the intervention.	_________4,5,9_ ___	______________
	**WHAT**		
**3.**	Materials: Describe any physical or informational materials used in the intervention, including those provided to participants or used in intervention delivery or in training of intervention providers. Provide information on where the materials can be accessed (e.g. online appendix, URL).	_______9_____	______________
**4.**	Procedures: Describe each of the procedures, activities, and/or processes used in the intervention, including any enabling or support activities.	_________9___	______________
	**WHO PROVIDED**		
**5.**	For each category of intervention provider (e.g. psychologist, nursing assistant), describe their expertise, background and any specific training given.	_______9_____	______________
	**HOW**		
**6.**	Describe the modes of delivery (e.g. face-to-face or by some other mechanism, such as internet or telephone) of the intervention and whether it was provided individually or in a group.	_______9_____	______________
	**WHERE**		

For peer review only

For peer review only - http://bmjopen.bmj.com/site/about/guidelines.xhtml

**7.**	Describe the type(s) of location(s) where the intervention occurred, including any necessary infrastructure or relevant features.	9	
	**WHEN and HOW MUCH**		
**8.**	Describe the number of times the intervention was delivered and over what period of time including the number of sessions, their schedule, and their duration, intensity or dose.	9	
	**TAILORING**		
**9.**	If the intervention was planned to be personalised, titrated or adapted, then describe what, why, when, and how.	9	
	**MODIFICATIONS**		
**10.‡**	If the intervention was modified during the course of the study, describe the changes (what, why, when, and how).	NA	
	**HOW WELL**		
**11.**	Planned: If intervention adherence or fidelity was assessed, describe how and by whom, and if any strategies were used to maintain or improve fidelity, describe them.		
**12.‡**	Actual: If intervention adherence or fidelity was assessed, describe the extent to which the intervention was delivered as planned.	NA	

For peer review only

** **Authors** - use N/A if an item is not applicable for the intervention being described. **Reviewers** – use '?' if information about the element is not reported/not sufficiently reported.

† If the information is not provided in the primary paper, give details of where this information is available. This may include locations such as a published protocol or other published papers (provide citation details) or a website (provide the URL).

‡ If completing the TIDieR checklist for a protocol, these items are not relevant to the protocol and cannot be described until the study is complete.

* We strongly recommend using this checklist in conjunction with the TIDieR guide (see *BMJ* 2014;348:g1687) which contains an explanation and elaboration for each item.

* The focus of TIDieR is on reporting details of the intervention elements (and where relevant, comparison elements) of a study. Other elements and methodological features of studies are covered by other reporting statements and checklists and have not been duplicated as part of the TIDieR checklist. When a **randomised trial** is being reported, the TIDieR checklist should be used in conjunction with the CONSORT statement (see www.consort-statement.org) as an extension of **Item 5 of the CONSORT 2010 Statement.** When a **clinical trial protocol** is being reported, the TIDieR checklist should be used in conjunction with the SPIRIT statement as an extension of **Item 11 of the SPIRIT 2013**

For peer review only - http://bmjopen.bmj.com/site/about/guidelines.xhtml

Statement (see www.spirit-statement.org). For alternate study designs, TIDieR can be used in conjunction with the appropriate checklist for that study design (see www.equator-network.org).

For peer review only

For peer review only - http://bmjopen.bmj.com/site/about/guidelines.xhtml
